# Genome-wide identification and expression analyses of the LEA protein gene family in tea plant reveal their involvement in seed development and abiotic stress responses

**DOI:** 10.1038/s41598-019-50645-8

**Published:** 2019-10-01

**Authors:** Xiaofang Jin, Dan Cao, Zhongjie Wang, Linlong Ma, Kunhong Tian, Yanli Liu, Ziming Gong, Xiangxiang Zhu, Changjun Jiang, Yeyun Li

**Affiliations:** 1 Fruit and Tea Research Institute, Hubei Academy of Agricultural Sciences, Wuhan, 430064 China; 2 State Key Laboratory of Tea Plant Biology and Utilization, Anhui Agricultural University, Hefei, 230036 China

**Keywords:** Genomic analysis, Gene expression analysis

## Abstract

Late embryogenesis abundant (LEA) proteins are widely known to be present in higher plants and are believed to play important functional roles in embryonic development and abiotic stress responses. However, there is a current lack of systematic analyses on the LEA protein gene family in tea plant. In this study, a total of 48 *LEA* genes were identified using Hidden Markov Model profiles in *C. sinensis*, and were classified into seven distinct groups based on their conserved domains and phylogenetic relationships. Genes in the CsLEA_2 group were found to be the most abundant. Gene expression analyses revealed that all the identified *CsLEA* genes were expressed in at least one tissue, and most had higher expression levels in the root or seed relative to other tested tissues. Nearly all the *CsLEA* genes were found to be involved in seed development, and thirty-nine might play an important role in tea seed maturation concurrent with dehydration. However, only sixteen *CsLEA* genes were involved in seed desiccation, and furthermore, most were suppressed. Additionally, forty-six *CsLEA* genes could be induced by at least one of the tested stress treatments, and they were especially sensitive to high temperature stress. Furthermore, it was found that eleven *CsLEA* genes were involved in tea plant in response to all tested abiotic stresses. Overall, this study provides new insights into the formation of *CsLEA* gene family members and improves our understanding on the potential roles of these genes in normal development processes and abiotic stress responses in tea plant, particularly during seed development and desiccation. These results are beneficial for future functional studies of *CsLEA* genes that will help preserve the recalcitrant tea seeds for a long time and genetically improve tea plant.

## Introduction

LEA proteins were first identified 30 years ago in cotton seeds during the late stages of embryo development^1^. Since this discovery, these proteins have also been detected in the roots, stems, leaves, flowers, and other tissues of plants^2–4^. LEA proteins are widely distributed in higher plants, such as *A. thaliana*^3^, *O. sativa*^5^, and *P. trichocarpa*^6^, and they are found in algae, fungi, bacteria^7^, and even invertebrates^8^. According to their distinct motif compositions, amino acid sequences, and phylogenetic relationships, LEA proteins have been classified into at least eight different groups (LEA_1, LEA_2, LEA_3, LEA_4, LEA_5, LEA_6 (PvLEA18), dehydrin (DHN), and seed maturation protein (SMP)) within the Pfam database^9^. However, currently no distinct universal classification criterion for LEA proteins has been established^10–12^. *LEA* genes often exist as a large gene family in higher plants. To date, the genome-wide identification and analysis of the LEA gene family has been performed in a few genome sequenced plant species, such as *A. thaliana*^3^, *O. sativa*^5^, *Z. mays*^13^, *S. tuberosum*^14^, *C. sativus*^15^, *S. lycopersicum*^16^, *B. napus*^4^, *M. esculenta*^17^, *P. trichocarpa*^6^, *P. mume*^18^, and *P. tabuliformis*^19^.

Most LEA proteins are highly hydrophilic and intrinsically disordered in their natural forms because they contain high percentages of charged amino acid residues, as well as glycine or other small amino acids (alanine, serine, and threonine), and they either lack or contain small amounts of cysteine and tryptophan residues^2,20,21^. However, some of these proteins may form three-dimensional structures to a degree during desiccation or under extreme temperature conditions^22^. LEA proteins are considered to play important roles in the normal growth and development of plants, as well as roles in mitigating the detrimental effects of various stress conditions in cells. These proteins were found to accumulate at high concentrations during the last period of seed maturation, concurrent with dehydration^1,23^. More importantly, it has been demonstrated that their expression may be significantly induced under abiotic stress conditions, such as cold, heat, and drought^15,24,25^. It has been proposed that LEA proteins may be involved in various important functions against abiotic stresses, including the stabilization of membrane structures^2,26,27^, the scavenging free radicals^28,29^, and the sequestering ions^29,30^ or biotin^31^ etc. At the cellular level, a subcellular location analysis revealed that LEA proteins are mainly located in the nucleus and the cytoplasm^32^.

Tea plant (*Camellia sinensis* (L.) O. Kuntze) is an important perennial woody crop used for the production of widely consumed non-alcoholic beverages, and is primarily grown in tropical and subtropical regions. However, tea plant is susceptible to various abiotic stresses, such as low temperature, high temperature, and drought, which seriously affect productivity and quality and restrict spatial distribution^33^. Meanwhile, tea seeds categorized as recalcitrant are highly sensitive to desiccation and cannot be used to preserve tea genetic resources for a long time^34^. Studies have shown the response of *CsLEA* genes to low temperature, drought, salinity, and desiccation stresses, which signifies their important roles in imparting stress tolerance^35–37^. Although 33 *LEA* genes have been identified based on the analysis of BLASTP searches against the tea plant genome^38^, our transcriptome analysis found additional *CsLEA* genes, which participated in the desiccation treatment process of recalcitrant tea seeds, had not been reported^37^. Furthermore, the biological functions of *CsLEA* genes that respond to high temperature and exogenous abscisic acid (ABA) stresses, particularly during seed development and desiccation in tea plant, remain unknown.

Therefore, in the present study, a comprehensive genome-wide identification of CsLEA protein genes was performed based on two tea plant genomes and three transcriptomes by Hidden Markov Model (HMM) profiles, and their sequence characteristics, phylogenetic relationships, conserved motifs, and gene structures were investigated. Meanwhile, the expression profiles of *CsLEA* genes in five different tissues, during the seed development process, during the seed desiccation process, and during responses to low temperature, high temperature, drought, and ABA stresses were analyzed. This systematic study provides new information on the LEA protein gene family in tea plant, and furthers our understanding of *CsLEA* genes associated with seed development, seed desiccation, and abiotic stress responses. Our findings will help in the genetic improvement of tea plant and contribute to the preservation of tea seeds as genetic resources for a long time.

## Materials and Methods

### Plant materials and stress treatments

The tea plant cultivar ‘*C. sinensis* cv. Echa 1’ was used in this study. To analyze the tissue-specific expression profiles of the identified *CsLEA* genes, the roots, stems, leaves, flowers, and seeds were sampled from tea plant grown in the experimental fields of Fruit and Tea Research Institute, Hubei Academy of Agricultural Sciences. To investigate the involvement of the *CsLEA* genes in tea seed development, seeds were collected in the morning at seven different developmental time points (April 30, May 31, June 30, July 31, August 31, September 30, and October 31 in 2017). To investigate the relationship between the *CsLEA* genes and tea seed desiccation, seeds were collected at four different desiccation time stages (0, 3, 5, and 8 d), following the method described in our previous study^37^. Two-year-old cutting seedlings planted in pots were placed in an artificial climate chamber (Yiheng, Shanghai, China), and maintained at a constant temperature of 25 ± 1 °C with a constant photoperiod for at least one week before the application of abiotic stress treatments. For high and low temperature treatments, the temperature of the artificial climate chamber was set to 38 °C or 4 °C, respectively, while maintaining all other growing conditions. For drought treatment, the tea seedlings were removed from the pots and carefully washed with distilled water to remove soil from the roots, and then transferred into 10% (w/v) polyethylene glycol 6000 (PEG 6000) solution. For ABA treatment, freshly prepared working solution of 100 µM exogenous ABA was sprayed on the leaves of tea seedlings. The second and/or third mature leaves from the shoot apexes were collected at 0, 6, 12, and 24 h during the previously described stress treatments and 0 h time point was used as the control. All samples were immediately frozen in liquid nitrogen and stored at −80 °C for subsequent gene expression analysis. Three independent biological replicates were conducted.

### Identification and characterization of *LEA* genes in *C. sinensis*

Hidden Markov Model profiles of LEA proteins with the accession numbers PF03760 (LEA_1), PF03168 (LEA_2), PF03242 (LEA_3), PF02987 (LEA_4), PF00477 (LEA_5), PF10714 (LEA_6), PF00257 (DHN), and PF04927 (SMP) were downloaded from the Pfam database (http://pfam.xfam.org/)^9^. All the putative *CsLEA* genes were obtained by searching two tea plant genomes (http://www.plantkingdomgdb.com/tea_tree/ ^39^ and http://tpia.teaplant.org)^40^ and three transcriptomes (SRP096975, SRP108833, and SRP124749) using HMMER 3.1 software (http://hmmer.org/). All the identified candidate genes were analyzed using the Pfam database (http://pfam.xfam.org/)^9^ and the NCBI Conserved Domain Search database (https://www.ncbi.nlm.nih.gov/Structure/cdd/wrpsb.cgi)^41^ to confirm the presence of LEA conserved domains. CsLEA proteins without conserved domains and a complete CDS sequence were manually removed. Redundant *CsLEA* genes were also discarded.

To investigate the characteristics of CsLEA proteins, the molecular weight (MW), theoretical isoelectric point (pI), and grand average of hydropathy (GRAVY) were predicted using the ProtParam tool (http://web.expasy.org/protparam/)^42^. Furthermore, the subcellular localization predictions for these CsLEA proteins were carried out using the WoLF PSORT tool (http://www.genscript.com/wolf-psort.html)^43^.

### Sequence alignment, phylogenetic analysis, and identification of gene structures and conserved motifs of CsLEA proteins

A multiple sequence alignment of all the identified CsLEA protein sequences and 51 AtLEA protein sequences^3^ (Table S1) was performed using ClustalX 2.1 software with the default parameters^44^. A phylogenetic analysis based on the amino acid sequences was constructed using MEGA 7.0 software with the 1000 bootstrapped Neighbor-Joining (NJ) method^45^. The exon-intron structure information of all the identified *CsLEA* genes from two tea plant genomes was obtained using GSDS 2.0 (Gene Structure Display Server, http://gsds.cbi.pku.edu.cn/)^46^. A protein conserved motif analysis was conducted using the MEME (Multiple Expectation Maximization for Motif Elicitation) Suite (http://meme-suite.org/)^47^. The parameters for motif identification were set as follows: maximum number of motifs, 10; site distribution, any number of repetitions; minimum motif width, 6; maximum motif width, 50.

### RNA isolation and qRT-PCR analysis

Total RNA was isolated from samples using an EASYspin Plus Complex Plant RNA Kit (Aidlab, Beijing, China) according to the manufacturer’s instructions. The concentration and quality of the RNA samples were assessed using a NanoDrop 2000 spectrophotometer (Thermo Scientific, Wilmington, DE, USA) and agarose gel electrophoresis, respectively. cDNA synthesis was completed using a PrimeScript™ RT Reagent Kit (TaKaRa, Dalian, China), and the specific primers were designed using Primer Premier 5.0 (Table S2). qRT-PCR analyses were performed using a BioRad CFX96 Real-Time PCR system (Bio-Rad, CA, USA). The reaction conditions of qRT-PCR were as follows: 30 s at 95 °C, followed by 45 cycles of 5 s at 95 °C and 30 s at 60 °C. Glyceraldehyde-3-phosphate dehydrogenase (GAPDH) gene was used as the internal control. The relative expression levels of these genes were calculated using the 2^−ΔCt^ or 2^−ΔΔCt^ method^48^. Three biological replicates and three technical replicates were performed.

### Determination of seed moisture content

The moisture content of seeds was determined gravimetrically by oven drying the seeds at 103 °C for 17 h^49^. Three replicates, containing 50 seeds each, were used to determine the seed moisture content expressed on a fresh mass basis.

### Statistical analysis

Statistical analysis of qRT-PCR and seed moisture content was performed using the SPSS 17.0 software. The data is presented as mean ± standard deviation (n = 3).

## Results

### Identification and characteristics of *CsLEA* genes in *C. sinensis*

Based on the tea plant genomes and three transcriptomes in available for this study, a total of 48 LEA protein genes, named as *CsLEA1* to *CsLEA48*, was identified in *C. sinensis* (Table 1). All these *CsLEA* genes contained full open reading frames, and could be classified into seven distinct groups using the Pfam family domain analysis^9^, including 2 in LEA_1, 32 in LEA_2, 4 in LEA_3, 2 in LEA_4, 1 in LEA_5, 4 in DHN, and 3 in SMP protein-encoding genes. However, no genes classified in the LEA_6 group were found. According to an analysis of physiochemical properties, the protein lengths of all the CsLEA proteins changed between 72 and 451 amino acids, and their molecular weights varied from 7.55 to 49.47 kDa. Their theoretical isoelectric points ranged from 10.16 to 4.72, where 36 of the proteins (75.0%) were considered to be basic (pI > 7) and 12 (25.0%) were considered to be acidic (pI < 7). Additionally, the calculated grand average of hydropathy values showed that LEA_2 group contained hydrophilic and hydrophobic proteins, while all the CsLEA proteins in the remaining groups were found to be highly hydrophilic. A subcellular localization prediction indicated that the majority of the CsLEA proteins were primarily localized to the cytoplasm, nucleus, and chloroplast, and all proteins in the LEA_4, LEA_5, and DHN groups were exclusively located in the nucleus.

**Table 1:** Characteristics of genes encoding LEA proteins in *C. sinensis*.

Name	Gene ID	Group	No. of domain	Protein Length (aa)	MW (kDa)	pI	GRAVY	Predicted subcellular localization	Arabidopsis ortholog
*CsLEA1*	CSA000844.1/TEA008269.1	LEA_2	1	231	26.03	9.39	0.015	Chloroplast	
*CsLEA2*	CSA000980.1/TEA029076.1	LEA_2	1	183	19.68	5.21	0.361	Chloroplast	
*CsLEA3*	CSA001281.1	LEA_2	1	229	25.23	9.30	0.176	Chloroplast	
*CsLEA4*	CSA002179.1/TEA012563.1	LEA_2	1	215	23.95	6.72	−0.032	Cytoplasmic	
*CsLEA5*	CSA002944.1/TEA018323.1*	LEA_2	1	206	22.68	9.71	0.197	Cytoplasmic	
*CsLEA6*	CSA003226.1/TEA005685.1*	LEA_2	1	197	21.50	9.37	0.287	Chloroplast	
*CsLEA7*	CSA003726.1*	LEA_3	1	96	10.47	9.99	−0.431	Cytoplasmic	AT4G02380
*CsLEA8*	CSA004403.1/TEA010666.1*	Dehydrin	2	254	29.01	5.29	−1.470	Nuclear	AT1G20440
*CsLEA9*	CSA006264.1/TEA013720.1	LEA_3	1	106	11.96	7.09	−0.782	Mitochondrial	
*CsLEA10*	CSA006938.1/TEA006416.1*	LEA_3	1	96	10.30	10.08	−0.467	Mitochondrial	AT4G02380
*CsLEA11*	CSA007094.1	LEA_4	1	278	30.58	5.11	−0.887	Nuclear	AT2G42560
*CsLEA12*	CSA009009.1*	LEA_1	1	106	11.39	7.11	−1.045	Mitochondrial	AT2G35300
*CsLEA13*	CSA009868.1/TEA030980.1*	LEA_2	1	210	23.57	9.92	−0.232	Cytoplasmic	
*CsLEA14*	CSA010151.1/TEA003016.1*	LEA_4	2	187	20.69	7.64	−0.987	Nuclear	AT3G53040
*CsLEA15*	CSA010673.1/TEA032971.1*	LEA_2	1	225	24.77	9.51	0.014	Cytoplasmic	
*CsLEA16*	CSA010820.1/TEA033104.1	LEA_2	1	248	27.19	9.79	−0.158	Chloroplast	
*CsLEA17*	CSA012431.1*	LEA_3	1	86	9.83	9.69	−0.385	Chloroplast	AT1G02820
*CsLEA18*	CSA013054.1/TEA014162.1	LEA_2	1	250	27.84	10.01	−0.156	Chloroplast	
*CsLEA19*	CSA013456.1/TEA033525.1	LEA_2	1	210	23.50	9.58	−0.156	Nuclear	
*CsLEA20*	CSA013879.1/TEA012020.1	LEA_2	1	196	22.11	9.85	−0.160	Mitochondrial	
*CsLEA21*	CSA016568.1/TEA015482.1	LEA_2	1	271	29.75	9.75	−0.062	Cytoplasmic	
*CsLEA22*	CSA019232.1*	SMP	1	72	7.55	5.62	−0.101	Cytoplasmic	AT3G22490
*CsLEA23*	CSA019926.1/TEA024719.1	LEA_2	1	451	49.47	8.81	−0.159	Chloroplast	
*CsLEA24*	CSA021165.1/TEA030404.1	LEA_2	1	228	25.71	9.23	−0.161	Chloroplast	
*CsLEA25*	CSA021249.1/TEA007147.1*	LEA_2	1	211	23.19	9.66	0.067	Chloroplast	
*CsLEA26*	CSA026452.1/TEA028236.1*	LEA_1	1	142	14.90	6.13	−0.544	Nuclear	AT5G06760
*CsLEA27*	CSA027135.1	LEA_2	1	211	23.51	9.41	−0.029	Plasma membrane	
*CsLEA28*	CSA027557.1/TEA031400.1*	SMP	2	213	22.27	4.72	−0.185	Chloroplast	AT3G22490
*CsLEA29*	CSA028043.1	LEA_2	1	249	28.38	9.36	−0.128	Cytoplasmic	
*CsLEA30*	CSA030792.1/TEA011337.1*	LEA_2	2	318	35.36	4.86	−0.371	Cytoplasmic	AT2G44060
*CsLEA31*	CSA030897.1/TEA008241.1	LEA_2	1	253	28.05	9.63	0.051	Cytoplasmic	
*CsLEA32*	CSA031822.1/TEA014379.1*	LEA_2	1	153	16.64	4.93	−0.008	Cytoplasmic	AT1G01470
*CsLEA33*	CSA032382.1/TEA014165.1	LEA_2	1	210	23.91	9.43	0.127	Cytoplasmic	
*CsLEA34*	CSA033045.1/TEA015292.1*	LEA_2	1	210	23.36	9.95	−0.111	Cytoplasmic	
*CsLEA35*	CSA034141.1/TEA032830.1*	LEA_2	1	221	24.55	10.16	0.010	Cytoplasmic	
*CsLEA36*	CSA035676.1*	LEA_5	1	78	8.59	5.45	−1.517	Nuclear	AT3G51810
*CsLEA37*	CSA036017.1/TEA021072.1*	LEA_2	1	194	21.29	10.02	−0.042	Cytoplasmic	
*CsLEA38*	NF	LEA_2	1	213	23.99	9.25	0.186	Cytoplasmic	
*CsLEA39*	NF	SMP	1	155	16.89	9.68	−0.741	Chloroplast	AT3G22490
*CsLEA40*	TEA005489.1	LEA_2	1	221	24.45	9.71	0.138	Cytoplasmic	
*CsLEA41*	NF	Dehydrin	1	208	21.70	6.95	−0.807	Nuclear	AT2G21490
*CsLEA42*	TEA007071.1	LEA_2	1	260	28.48	10.09	−0.149	Chloroplast	
*CsLEA43*	NF	LEA_2	1	208	23.25	9.32	0.202	Cytoplasmic	
*CsLEA44*	TEA018923.1	LEA_2	1	215	24.00	9.62	0.175	Cytoplasmic	
*CsLEA45*	TEA015290.1*	LEA_2	1	212	23.82	9.74	−0.263	Cytoplasmic	
*CsLEA46*	NF	Dehydrin	1	169	16.91	8.81	−1.050	Nuclear	AT5G66400
*CsLEA47*	TEA024718.1	LEA_2	1	262	28.45	9.41	−0.154	Chloroplast	
*CsLEA48*	NF	Dehydrin	1	210	21.98	6.90	−1.328	Nuclear	AT5G66400

Note: NF indicates these *CsLEA* genes were not found in two tea plant genomes, and asterisk (*)indicates these *CsLEA* genes were ever reported in the previously study^38^.

### Phylogenetic, gene structure and conserved motif analyses of *CsLEA* genes

**Figure 1:**
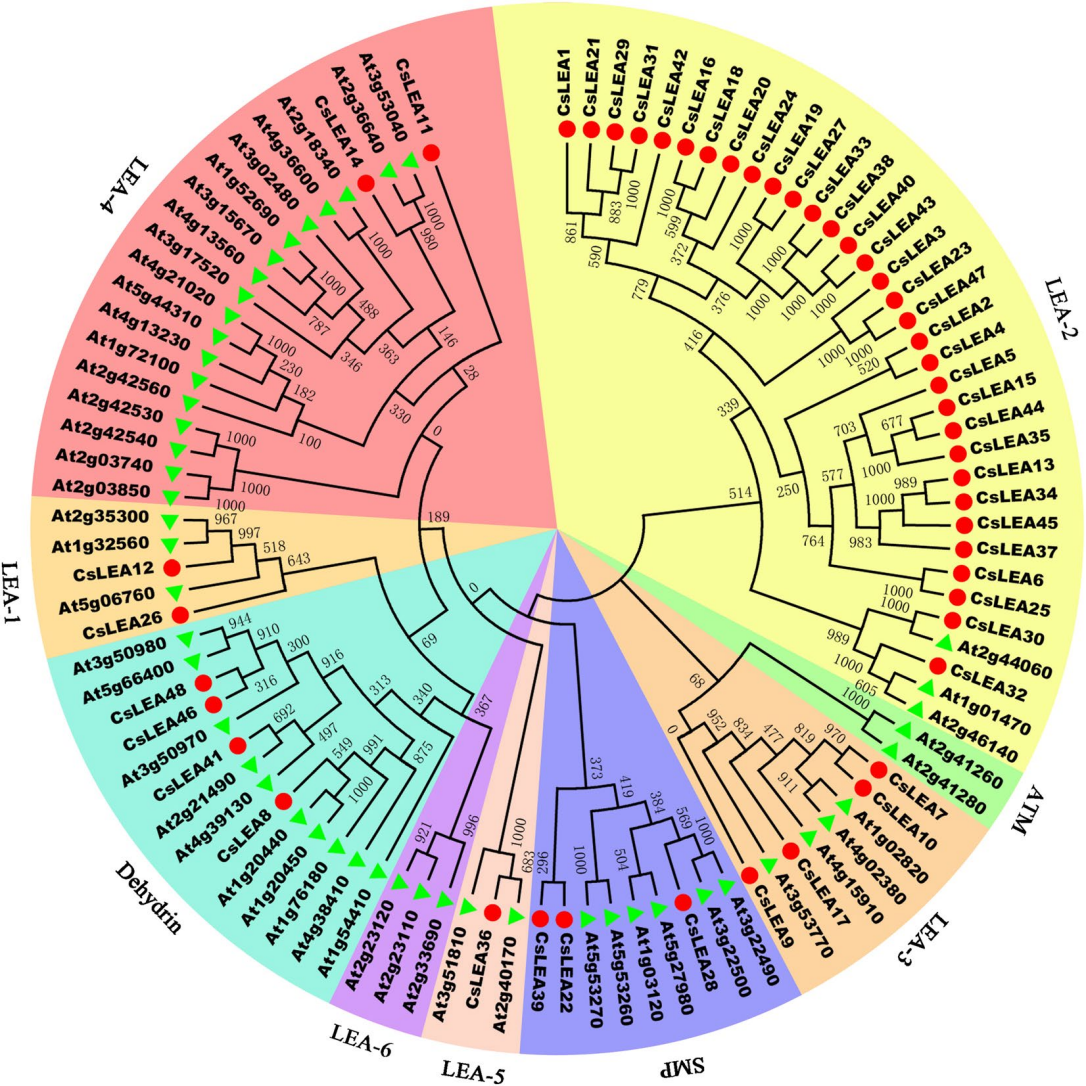
Phylogenetic relationships of the 48 *CsLEA* genes and 51 *AtLEA* genes. The phylogenetic tree was constructed using MEGA 7.0, and the nine major groups are marked with different color backgrounds.

A phylogenetic analysis, based on the amino acid sequences of the predicted proteins, revealed that the 48 *CsLEA* genes were clustered into seven distinct subfamilies together with *AtLEA* genes, which further confirmed that they belong to the LEA family (Fig. 1). One orthologous gene pair (CsLEA30 and At2g44060) was identified in *A. thaliana* and *C. sinensis* with relatively high bootstrap support (>97%). Nine paralogous gene pairs (CsLEA6 and CsLEA25, CsLEA7 and CsLEA10, CsLEA13 and CsLEA34, CsLEA16 and CsLEA18, CsLEA19 and CsLEA27, CsLEA23 and CsLEA47, CsLEA29 and CsLEA31, CsLEA33 and CsLEA38, and CsLEA40 and CsLEA43) identified in *C. sinensis* also had relatively high bootstrap support (>97%). Additionally, *CsLEA8*, *CsLEA14*, *CsLEA28*, and *CsLEA30* contained two LEA domains, while the remainder of the *CsLEA* genes had only a single LEA domain (Table 1).

Meanwhile, the exon-intron structure of *CsLEA* genes was analysed (Fig. S1). The majority of *CsLEA* genes contained no intron or 1 intron, except that five *CsLEA* genes contained 2 introns or 3 introns. Additionally, the LEA_5 group contained no intron, the LEA_3 and DHN groups contained only 1 intron, and the other groups contained 0–3 introns. Generally, CsLEA genes in the same group showed similar exon-intron structure, which evidences their phylogenetic relationships and the classification of groups.

**Figure 2:**
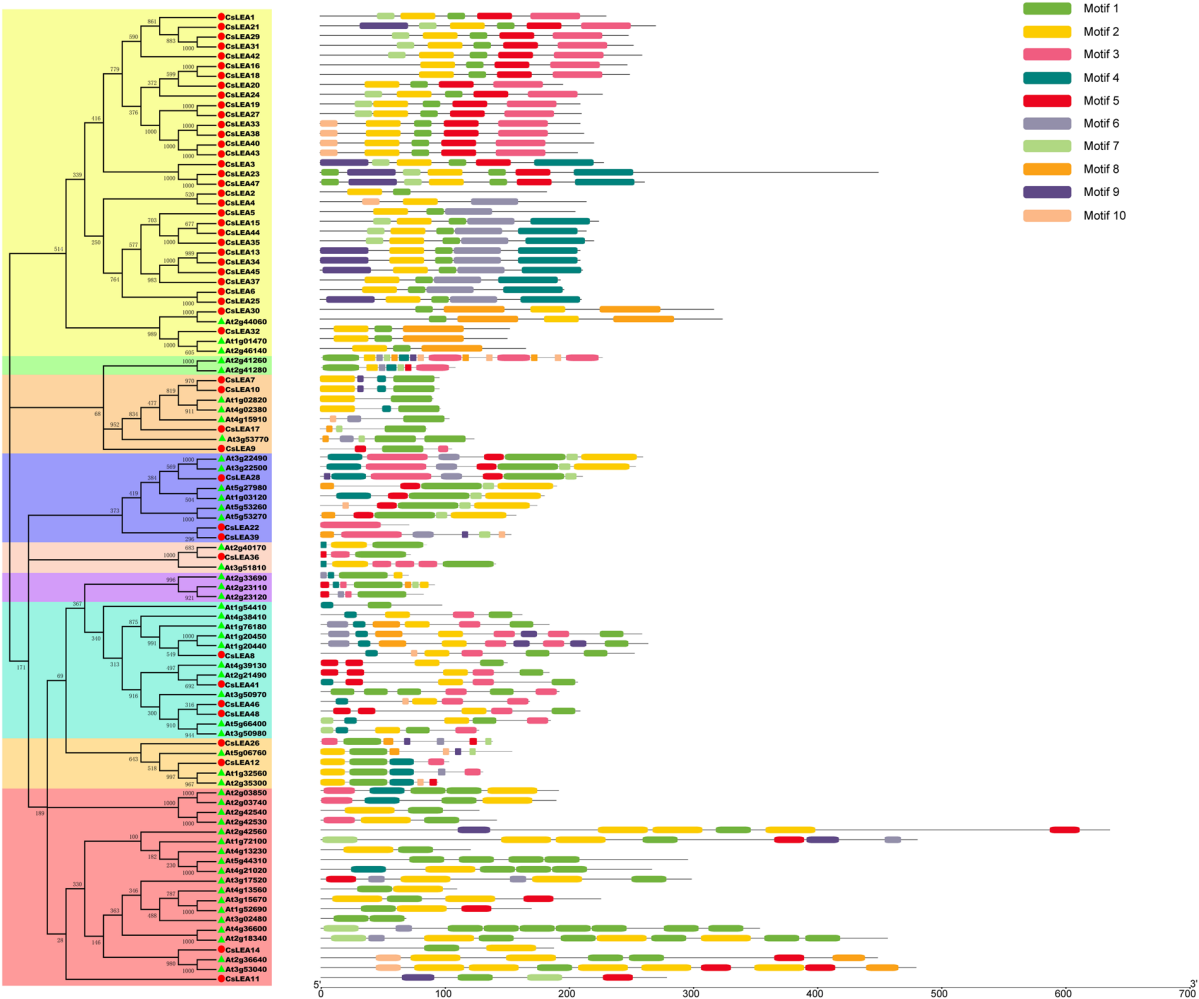
Phylogenetic relationships and motif compositions of the *LEA* genes in *C. sinensis* and *A. thaliana*. The phylogenetic tree on the left side was constructed using MEGA 7.0. The nine major groups are marked with different color backgrounds. The conserved motifs of each group on the right side were identified by the MEME web server. Different motifs are represented by different colored boxes, and the motif sequences are provided in Table S3.

To better understand the structural features of the CsLEA proteins, the conserved motifs were investigated using the MEME web server^46^. Since the 48 CsLEA proteins did not share high similarity, the seven subfamilies were respectively submitted to MEME and a total of ten conserved motifs were identified (Fig. 2). Results showed that the members of each LEA subfamily possess several group-specific conserved motifs (Table S3) that have been previously reported in other plant species (e.g., *A. thaliana*^3^, *B. napus*^4^, and *M. esculenta*^17^). For example, an important conserved motif, termed K segment for its richness in lysine (K) residues, was only identified in all four of the CsLEA proteins of the DHN group, which was the most characterized LEA group. Furthermore, the repetitions of this motif are variable in *C. sinensis* dehydrin proteins: twice in *CsLEA41*, *CsLEA46*, and *CsLEA48*, and up to three times in *CsLEA8*. Additionally, the S (a serine (S)-rich motif) and Y segments were also only observed in these four dehydrins. These results suggested that the composition of structural motifs varies among the different CsLEA groups, but is similar within a group, which further supports the proposed phylogenetic relationships of the CsLEA proteins. Furthermore, variations in the motif structures of the CsLEA proteins may indicate functional divergence.

### Expression profile analysis of *CsLEA* genes in five different *C. sinensis* tissues

**Figure 3:**
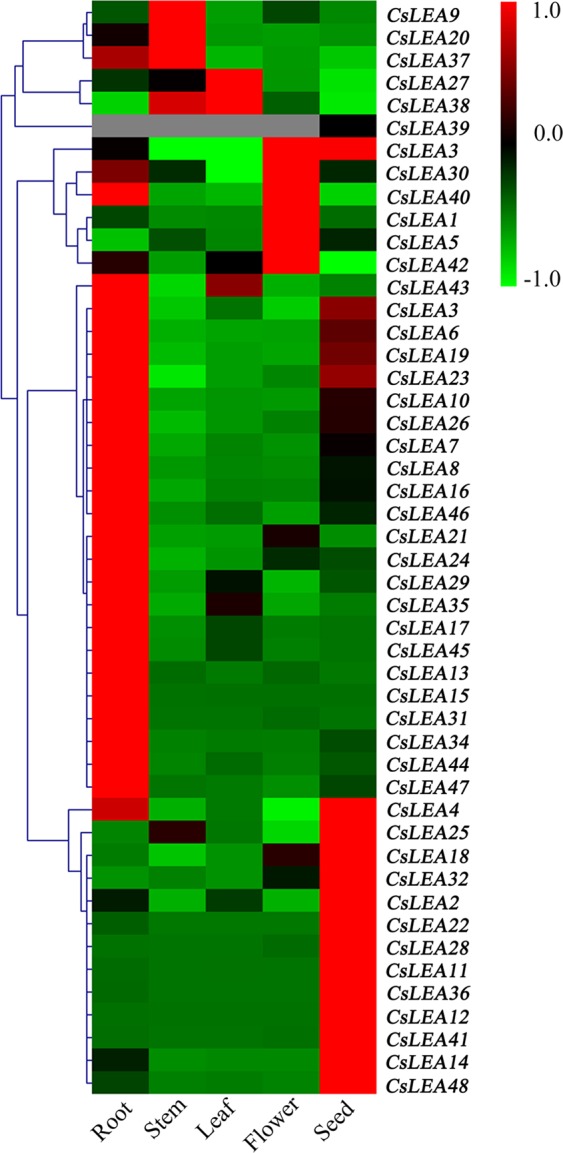
A heatmap showing the hierarchical clustering of the expression levels of the 48 *CsLEA* genes in the roots, stems, leaves, flowers, and seeds of tea plant. Relative expression values were calculated using the 2^−ΔCt^ method with GAPDH as a housekeeping gene. The heatmap was generated by MeV 4.9.0 software.

To investigate the tissue-specific expression profiles of the 48 *LEA* genes of tea plant, the expression levels of these genes in roots, stems, leaves, flowers, and seeds were determined using a qRT-PCR analysis. As shown in Fig. 3 and Table S4, with the exception of *CsLEA39* that specifically expressed in the seed, all other *CsLEA* genes were ubiquitously expressed in the five tissues and showed varying expression levels. Twenty-four of the *CsLEA* genes (3, 6, 7, 8, 10, 13, 15, 16, 17, 19, 21, 23, 24, 26, 29, 31, 34, 35, 40, 43, 44, 45, 46, and 47) were shown to be expressed at their highest levels in the root. *CsLEA9*, *CsLEA20*, and *CsLEA37* showed their highest expression levels in the stem. *CsLEA27* and *CsLEA38* showed their highest expression levels in the leaf. Five *CsLEA* genes (1, 5, 30, 33, and 42) showed their highest expression levels in the flower. Fourteen *CsLEA* genes (2, 4, 11, 12, 14, 18, 22, 25, 28, 32, 36, 39, 41, and 48) showed their highest expression levels in the seed. However, some of these genes (e.g., *CsLEA33* and *CsLEA40*) were also significantly expressed in other tissues. Generally, the majority of all the *CsLEA* genes had higher expression levels in the root or the seed than in any of the other tested tissues. Interestingly, the expression levels of *CsLEA22*, *CsLEA28*, and *CsLEA39*, which belonged to SMP subfamily, were the highest in the seed. Overall, the various expression levels of the *CsLEA* genes in the five different tissues reflect the diverse and important roles that they may play in the growth and development processes of tea plant.

### Expression profile analysis of *CsLEA* genes during the seed development process

**Figure 4:**
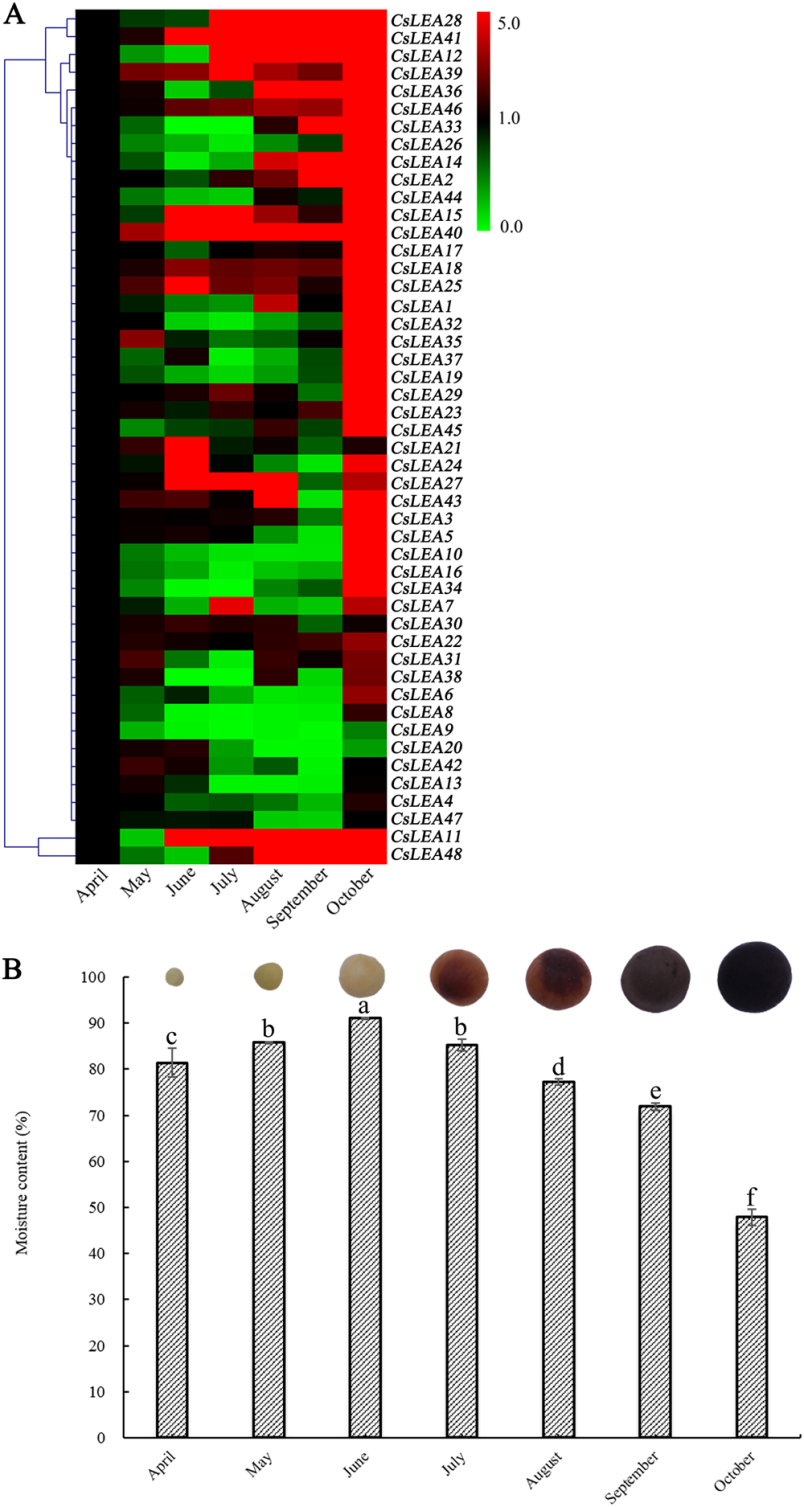
A heatmap showing the hierarchical clustering of the expression levels of the 48 *CsLEA* genes (**A**) and the changes in seed moisture content expressed as fresh weight (**B**) during seed development in tea plant. Relative expression values were calculated using the 2^−ΔΔCt^ method with GAPDH as a housekeeping gene. The heatmap was generated by MeV 4.9.0 software.

To further elucidate the relationship between *CsLEA* genes and seed development in tea plant, the expression levels of these genes at seven different developmental time points (April, May, June, July, August, September, and October) were detected using a qRT-PCR analysis. As shown in Fig. 4A and Table S5, the expression levels of *CsLEA39* and *CsLEA40* were significantly up-regulated throughout the entire seed development process. Among them, *CsLEA39* had its highest expression level in October, while *CsLEA40* peaked in August. Nine of the *CsLEA* genes (2, 3, 17, 18, 22, 23, 28, 41, and 46) remained at relatively stable expression levels in the early stages, and then their expression levels significantly increased in the late stages, reaching a peak in October. The expression levels of *CsLEA11* and *CsLEA12* were significantly down-regulated in May or/and June, and then gradually increased, at least doubling, in the subsequent developmental stages, where they achieved their maximum expression levels in October. Twenty-six of the *CsLEA* genes (1, 5, 6, 7, 10, 14, 15, 16, 19, 24, 25, 26, 27, 29, 31, 32, 33, 34, 35, 36, 37, 38, 43, 44, 45, and 48) had no obvious regularity in their expression levels from May to September, but their expression levels all increased more than two-fold in October. Conversely, the expression levels of seven of the *CsLEA* genes (4, 8, 9, 13, 21, 42, and 47) were significantly induced in several months during the early stages, but their expression levels did not significantly change in October. The expression of the *CsLEA20* gene remained constant until June, and then its expression level was decreased by at least two-fold. The expression of *CsLEA30* had no detectable response within the time of seed development. These results indicated that almost all of the identified *CsLEA* genes participate, either directly or indirectly, in seed development. Additionally, changes in the moisture content of tea seeds during their development was also investigated (Fig. 4B). Results showed that the moisture content of the seeds slightly increased from April to June, and then gradually decreased in the subsequent developmental stages, which revealed that tea seeds became dehydrated in the later developmental stages.

### Expression profile analysis of *CsLEA* genes during the seed desiccation process

**Figure 5:**
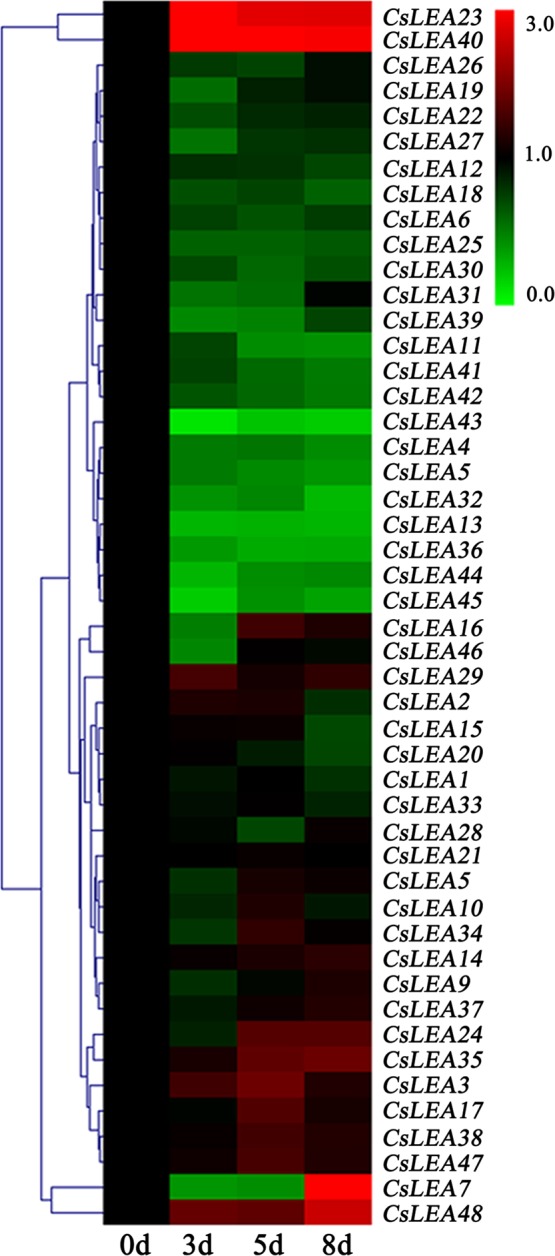
A heatmap showing the hierarchical clustering of the expression levels of the 48 *CsLEA* genes during seed desiccation process in tea plant. Relative expression values were calculated using the 2^−ΔΔCt^ method with GAPDH as a housekeeping gene. The heatmap was generated by MeV 4.9.0 software.

To further elucidate the relationship between *CsLEA* genes and tea seed desiccation, the expression levels of these genes at four different desiccation time stages (0, 3, 5, and 8 days) were detected using a qRT-PCR analysis. As shown in Fig. 5 and Table S6, two of the *CsLEA* genes were induced, showing significantly up-regulated expression during the entire seed desiccation process, and *CsLEA23* had its highest expression level at 3 d, while *CsLEA40* peaked at 5 d. *CsLEA48* remained at a relatively stable expression level until 5 d, then its expression level significantly increased, peaking at 8 d. The expression levels of *CsLEA7* were significantly down-regulated at 3 d and 5 d, but were subsequently increased more than two-fold at 8 d. Conversely, the expression levels of six of the *CsLEA* genes (13, 32, 36, 43, 44, and 45) were suppressed by at least two-fold over the entire seed desiccation process, and the expression levels of six of the *CsLEA* genes (4, 5, 11, 16, 39, and 46) were significantly inhibited various at individual time points. Additionally, the remaining thirty-two *CsLEA* genes showed no significant changes in their expression levels in response to seed desiccation. These results revealed that some of the *CsLEA* genes actively involved in the tea seed desiccation process.

### Expression profile analysis of *CsLEA* genes in response to low and high temperature stresses

**Figure 6:**
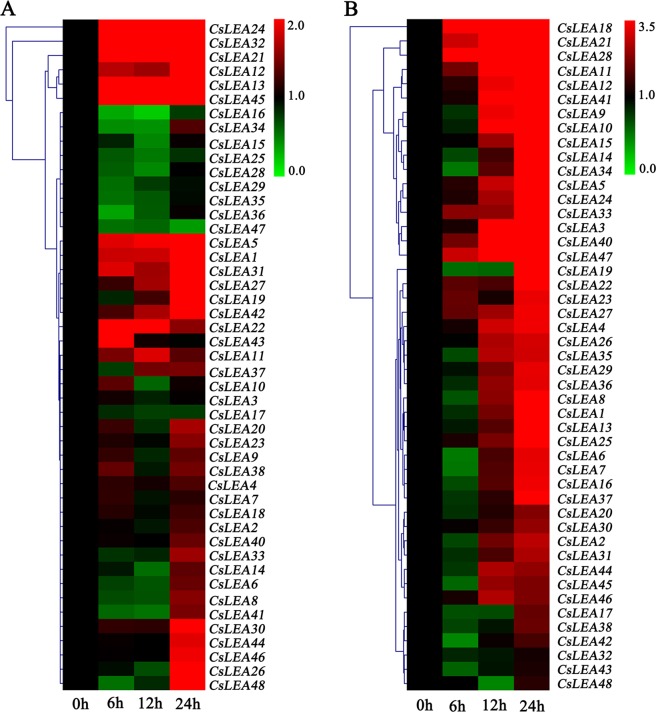
A heatmap showing the hierarchical clustering of the expression levels of the 47 *CsLEA* genes in response to various temperature stresses. (A) Low temperature (4 °C) stress. (B) High temperature (38 °C) stress. Relative expression values were calculated using the 2^−ΔΔCt^ method with GAPDH as a housekeeping gene. The heatmap was generated by MeV 4.9.0 software.

To investigate the responses of 47 *CsLEA* genes (except for *CsLEA39* that specifically expressed in the seed) to low and high temperature stresses, the expression levels of these genes under short-term low and high temperature stresses (0, 6, 12, and 24 h) were detected using a qRT-PCR analysis. Results revealed that the *CsLEA* genes are differentially expressed under low and high temperature stresses (Fig. 6). Under low temperature (4 °C) stress, five of the *CsLEA* genes were induced to show significantly up-regulated expression throughout the entire time course (Fig. 6A and Table S7). The highest expression levels of *CsLEA13*, *CsLEA32*,and *CsLEA45* were observed at 24 h, while *CsLEA21* and *CsLEA24* were dramatically induced at 6 h. Ten of the *CsLEA* genes (1, 5, 12, 19, 26, 27, 30, 31, 42, and 48) exhibited relatively stable expression levels until 12 h, and then their expression levels began to significantly rise, reaching their the highest levels at 24 h. The expression levels of *CsLEA22* and *CsLEA43* were up-regulated by at least two-fold during the early time points, but were not significantly affected at 24 h. The expression levels of six of the *CsLEA* genes (15, 16, 28, 34, 36, and 47) were significantly suppressed at individual time points, while no significant changes were seen in any of the other genes. Conversely, the trends in the expression levels of the *CsLEA* genes under high temperature stress (38 °C) were relatively obvious (Fig. 6B and Table S8). The expression levels of seven of the *CsLEA* genes (11, 18, 21, 28, 33, 40, and 47) gradually increased by at least two-fold, reaching their maximum levels at 24 h. Thirty-five of the *CsLEA* genes were stably expressed during the early time points, then their expression levels significantly increased in the late time points, peaking at 12 h or 24 h. Although high temperature stress positively regulated the transcription of most of the *CsLEA* genes, a few genes, such as *CsLEA42* and *CsLEA48*, were significantly suppressed at individual time points. Additionally, the expression levels of *CsLEA17*, *CsLEA32*, and *CsLEA43* were not significantly affected under high temperature stress. Overall, the trends in the expression levels of most of the *CsLEA* genes differed under low temperature and high temperature stress, but similar responses to both temperature stresses were observed for a few genes (e.g., *CsLEA19* and *CsLEA21*). These results indicated that the expression of the different *CsLEA* genes under temperature stresses were diverse and complex.

### Expression profile analysis of *CsLEA* genes in response to drought and ABA stresses

**Figure 7:**
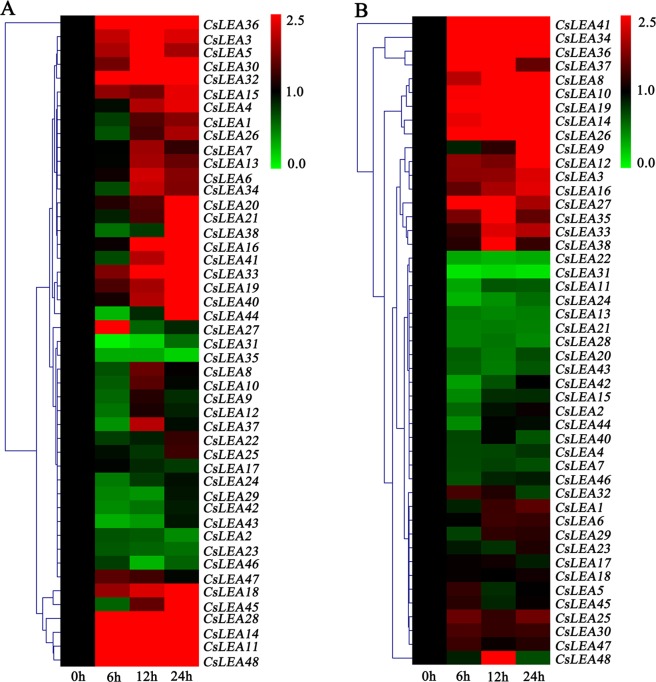
A heatmap showing the hierarchical clustering of the expression levels of the 47 *CsLEA* genes in response to drought (**A**) and ABA (**B**) stresses. Relative expression values were calculated using the 2^−ΔΔCt^ method with GAPDH as a housekeeping gene. The heatmap was generated by MeV 4.9.0 software.

To explore the putative functions of 47 *CsLEA* genes under drought (PEG 6000) and ABA stresses, their dynamic responses at four different time points were analyzed using a qRT-PCR analysis. Under PEG stress, the expression profiles of the *CsLEA* genes were complex (Fig. 7A and Table S9). The expression levels of seven of the *CsLEA* genes were significantly up-regulated at all treatment time points compared with the control. Among these genes, *CsLEA3*, *CsLEA11*, *CsLEA32*, and *CsLEA36* showed their highest expression levels at 12 h, while *CsLEA14*, *CsLEA28*, and *CsLEA48* peaked at 24 h. Thirteen of the *CsLEA* genes (4, 15, 16, 18, 19, 20, 21, 30, 33, 38, 40, 41, and 45) were stably expressed at the early time points, and then their expression levels significantly increased in the late time points, peaking at 24 h. The expression levels of four of the *CsLEA* genes (5, 6, 27, and 34) were significantly up-regulated at individual time points during the early stages, but their expression levels did not significantly change at 24 h. Conversely, the expression of *CsLEA35* was significantly inhibited during the 24 h treatment period, and the expression levels of six of the *CsLEA* genes (2, 29, 31, 42, 43, and 46) were significantly suppressed at individual time points. Strangely, the expression levels of *CsLEA37* and *CsLEA44* initially significantly decreased to their lowest levels at 6 h, and then increased by at least two-fold at 12 h and 24 h, respectively. Additionally, the expression levels of the fourteen remaining *CsLEA* genes showed no significant changes under drought stress treatment. Under exogenous ABA stress, the *CsLEA* genes were expressed to various degrees (Fig. 7B and Table S10). The expression levels of eight of the *CsLEA* genes were significantly increased at all the treated time points, where *CsLEA34* and *CsLEA36* peaked at 12 h, and *CsLEA8*, *CsLEA10*, *CsLEA14*, *CsLEA19*, *CsLEA26*, and *CsLEA41* peaked at 24 h. The expression levels of five of the *CsLEA* genes (3, 9, 12, 16, and 33) were relatively stable during the early time points, and then their expression levels significantly increased, reaching their highest levels at 12 h or 24 h. The expression levels of five of *CsLEA* genes (27, 35, 37, 38, and 48) were significantly up-regulated at 6 h and/or 12 h, and were subsequently decreased to the level of the control. Comparatively, the expression levels of *CsLEA22* and *CsLEA31* were down-regulated by at least two-fold during the treatment time, and the expression levels of eight of the *CsLEA* genes (11, 13, 15, 21, 24, 28, 42, and 44) were significantly decreased at individual time points. Furthermore, the expression levels of the remainder of the *CsLEA* genes showed no significant changes in response to ABA stress. Overall, these results revealed that most of the *CsLEA* genes actively involved in the responses of tea plant to drought and ABA stresses, and the response mechanisms in which they are involved are diverse and complex.

## Discussion

Genes that encode LEA proteins are not only widely distributed across the plant kingdom, but they are also found in fungi, bacteria, and even animal kingdom^3,7,8,19,25^. Due to the important roles of LEA proteins in embryonic development and abiotic stress responses, they have been identified and characterized in some plant species^3,4,6,14,17^, including *C. sinensis*^38^. Although 33 *CsLEA* genes were previously discovered in *C. sinensis* based on its genome, no systematic analysis of the LEA protein gene family has been completed. Because these 33 genes were identified only by BLASTP searches, which used the LEA protein sequences of *A. thaliana* and *O. sativa* as queries against two *C. sinensis* genomes^39,40^, some CsLEA proteins may have been overlooked. Therefore, additional searches for genes that encode CsLEA proteins in two *C. sinensis* genomes and three transcriptomes were conducted using HMM profiles. As a result, a total of 48 *CsLEA* genes were identified in *C. sinensis*, which was more than those *CsLEA* members identified in a previous study^38^. Among these 48 genes, only twenty-one *CsLEA* genes were found to be identical, while the remaining genes were unique. Thus, the results of our study provides more information on the members of the LEA protein gene family in tea plant. Additionally, the results of the current study are consistent with the number of LEA genes found in *A. thaliana* (51)^3^, *C. songorica* (44)^25^, and *P. trichocarpa* (53)^6^, etc., and more than those found in *M. esculenta* (26)^17^, *P. tabuliformis* (23)^19^, and *S. lycopersicum* (27)^16^, etc., but much less than those found in *B. napus* (108)^4^, *G. hirsutum* (242)^50^, and *S. Tuberosum* (74)^14^, etc.

Based on the conserved domain and phylogenetic tree analyses, the 48 *CsLEA* genes were only divided into seven distinct groups, where the LEA_6 and AtM groups were not found, which was consistent with the results of *C. sinensis* from Wang *et al*.^38^. Interestingly, the LEA_6 group is also absent in a few higher plant species, such as *D. officinale*^51^ and *S. lycopersicum*^16^. Furthermore, the AtM group has only been found in *A. thaliana*^3^. This finding indicated that variation exists in the LEA protein gene family groups in some plant species. Additionally, the results of this study showed that *CsLEA* genes were mainly distributed in the LEA_2 group, which accounted for 66.7% of the LEA gene family members. More significantly, such a large proportion of the LEA_2 group has not been observed in the previous studies on *A. thaliana* (5.9%)^3^, *C. songorica* (13.6%)^25^, *M. esculenta* (15.4%)^17^, *P. tabuliformis* (4.3%)^19^, and *P. trichocarpa* (7.5%)^6^. This difference may be attributed to the improvement of plant genome annotations and the fact that the LEA_2 group is an atypical LEA protein group because these proteins are typically more hydrophobic^50^. These findings indicated that the LEA protein gene family in higher plants may be larger and much more complex than previously described.

Based on an analysis of physiochemical properties, it was found that most of the *CsLEA* genes encode relatively small proteins, in which 95.8% are less than 35 kDa, which was consistent with the findings of Chen *et al*. in *S. Tuberosum* (94.6%)^14^ and Liang *et al*. in *B. napus* (90.7%)^4^. Previous studies have shown that most LEA protein members were basic in nature^15,52^, which is consistent with the results of this current study. Furthermore, these results show that all CsLEA proteins are hydrophilic, with the exception of those in LEA_2 group that contains hydrophobic proteins. Similar characteristics have also been reported for LEA proteins in *A. thaliana*^3^, *P. trichocarpa*^6^, and *S. Tuberosum*^14^. This indicated that LEA proteins possessed apparently hydrophilic characteristics and are evolutionary conserved proteins in higher plants, which makes them to be totally or partially disordered, and allows them to act as molecular chaperones that contribute to the protection of plants from desiccation^53–55^. Subcellular localization analyses showed that the CsLEA proteins could be present in the cytoplasm, nucleus, chloroplast, and mitochondria, which was also reported for LEA proteins found in *D. officinale*^51^ and *S. bicolor*^52^. It can be inferred that CsLEA proteins have a ubiquitous distribution across subcellular compartments, which highlights the requirement for each cellular compartment to be provided with protective mechanisms during abiotic stresses^32^. Additionally, this analysis revealed that each CsLEA group contained conserved motifs that have been previously reported in other plant species^4,14,50^, which were similar within the same group, but varied greatly among the different groups. This indicated that *CsLEA* genes may encode functional LEA proteins that have group-specific functions, and members of the same CsLEA protein group may have originated from gene expansion within a group, while members of the different groups may be attributed to the evolution of groups from different ancestors.

Because gene expression analyses can provide valuable clues for gene functions^56,57^, the expression levels of the *CsLEA* genes in different tissues, during seed development, during seed desiccation, and under abiotic stresses (low temperature, high temperature, drought, and ABA) were investigated. In the present study, it was found that all the *CsLEA* genes were expressed in at least one tissue, which was consistent with previous observations of Cao *et al*. in *S. lycopersicum*^16^ and Pedrosa *et al*. in *C. sinensis*^55^, indicating that these genes are widely involved in normal growth and development of *C. sinensis*. Furthermore, these results showed that the *CsLEA* genes have higher expression levels in certain tissues, especially in the root and in the seed, which implies that they have functional diversity. Similar expression patterns were also observed in the LEA gene family in other plant species^14,17,52^ and in other gene families of *C. sinensis*^58,59^. Additionally, it was found that all the *CsLEA* genes that belong to the SMP group showed higher expression levels in the seed tissue, which suggested that this group has an important role in reproductive development.

Several previous studies have shown that genes that encode LEA proteins participated in the plant seed development process concurrent with maturation dehydration^1,2,51^. In this study, it was found, with the exception of *CsLEA30*, that all the *CsLEA* genes were widely expressed throughout the entire seed development process of *C. sinensis*. Among these genes, a few (e.g., *CsLEA21* and *CsLEA24*) showed abundant accumulation in the early period of seed development, which indicated that they were involved in the early seed growth. More importantly, thirty-nine (81.3%) of the *CsLEA* genes were shown to be highly accumulated in the latter stages of seed maturation, where some of them (e.g., *CsLEA11* and *CsLEA28*) were expressed more than thousand fold compared with the control, which was consistent with the findings of previous studies^4^. This indicated that these genes involved in the endosperm and late seed development. It was also found that tea seeds became dehydrated during the last period of seed development, especially in October, and the moisture content of the seed decreased sharply from 71.9% to 47.9%. These results implied that thirty-nine genes that encode CsLEA proteins are likely to have important roles in tea seed maturation concurrent with dehydration. Additionally, some studies have revealed that LEA proteins are associated with seed desiccation tolerance^60,61^. In the current study, only two genes that encode LEA proteins were found to be significantly up-regulated, while relatively more *CsLEA* genes were found to be significantly down-regulated during the entire seed desiccation process, which is similar to our previous report^37^. This suggested that the *CsLEA* genes may not play an important protective role in tea seeds in response to desiccation treatment.

**Figure 8:**
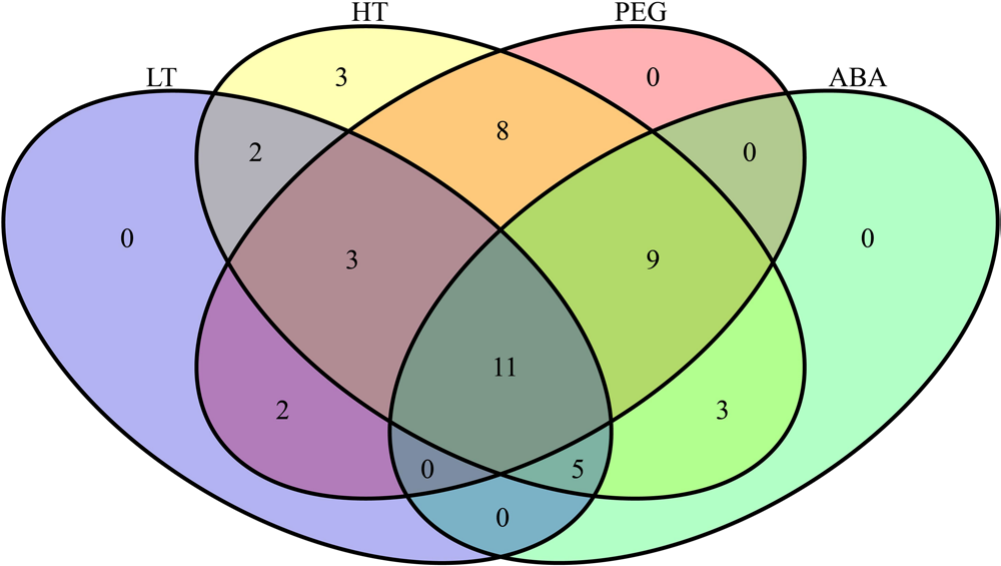
Venn diagram showing the number of *CsLEA* genes that responded to low temperature (LT), high temperature (HT), drought (PEG), and ABA stresses.

Several studies have demonstrated that genes that encode LEA proteins widely participate in a plant’s response to abiotic stresses, including cold^36^, heat^62^, drought^63,64^ and ABA^17^. In this study, nearly all of the identified *CsLEA* genes could be induced by at least one stress treatment, where twenty-three (48.9%), forty-four (93.6%), thirty-three (70.2%), and twenty-eight (59.6%) of the *CsLEA* genes were induced by low temperature, high temperature, drought, and ABA stresses, respectively. These results indicated that these genes play diverse roles in the regulation of tea plant acclimation to various abiotic stresses, and are highly sensitive to high temperature stress. Furthermore, it was found that eleven *CsLEA* genes were involved in the responses of tea plant to all the abiotic stresses assessed (Fig. 8), and fourteen *CsLEA* genes were involved in the responses to low temperature, high temperature, and drought stresses, which revealed that a single *CsLEA* gene participate in multiple stress responses. Interestingly, *CsLEA17* did not respond to any stress treatment applied in this study, but it was significantly expressed in the latter period of seed development, which suggested that this gene may be critical for seed maturation, but not for abiotic stress responses. Additionally, the expression of *CsLEA32*, *CsLEA36*, and *CsLEA45* were inhibited by at least two-fold during the entire seed desiccation process, while they were significantly up-regulated in the latter stages of seed maturation and responded to drought stress. Therefore, it was speculated that the repression of the expression of these three *CsLEA* genes may be an important cause of desiccation sensitivity in recalcitrant tea seeds.

Additionally, some studies have shown that the different LEA groups represented diversified adaptations to abiotic stresses in different plant species. For example, in *A. thaliana*, the LEA_3, LEA_4 and DHN groups could be induced by drought and salt stresses, while the LEA_1 group and the LEA_5 group were induced only by drought stress and salt stress, respectively^3^. However, in *P. tabuliformis*, all LEA groups could be induced by heat and salt stresses^19^. In the present study, the CsLEA_2, CsLEA_5, CsDHN, and CsSMP groups were shown to be regulated by all the assessed abiotic stresses, while there were no obvious responses of the CsLEA_1 group to drought, the CsLEA_3 group to low temperature and drought, and the CsLEA_4 group to low temperature. It was also observed that there was a clear divergence in expression profiles between genes within a single CsLEA group, with this phenomenon being especially apparent in the CsLEA_2 group. Overall, the results of this study revealed the functional roles of *LEA* genes in *C. sinensis* during seed development and desiccation and under varying abiotic stresses. Of course, additional studies are necessary to further elucidate and confirm the functions of these *CsLEA* genes.

## Conclusions

In conclusion, a total of 48 *CsLEA* genes were identified in *C. sinensis* and were subsequently classified into seven distinct groups, according to their conserved domains and phylogenetic relationships. Analyses of the physicochemical properties and conserved motifs of the CsLEA proteins showed that within the same groups they were highly similar, but the showed large variation between different groups. Additionally, the expression profiles of all the identified *CsLEA* genes in five different tissues, during seed development, during seed desiccation, and under four abiotic stresses, were further analyzed and revealed that these genes widely participate in tea plant’s growth, development, and responses to abiotic stresses, especially during seed development and under high temperature stress. Furthermore, eleven of these *CsLEA* genes were found to be involved in the response of tea plant to all the tested abiotic stresses. These results provide valuable information for the future functional studies of *CsLEA* genes, which will be useful for the preservation of recalcitrant tea seeds as genetic resources for a long time and the genetic improvement of tea plant.

## Supplementary information

Supplementary Figure S1

Supplementary Table S1

Supplementary Table S2

Supplementary Table S3

Supplementary Table S4

Supplementary Table S5

Supplementary Table S6

Supplementary Table S7

Supplementary Table S8

Supplementary Table S9

Supplementary Table S10

## Data Availability

All data supporting the findings of this study are available within this manuscript and its supplementary tables.

## References

[1] Dure L, Greenway SC, Galau GA (1981). Developmental biochemistry of cottonseed embryogenesis and germination: changing messenger ribonucleic acid populations as shown by *in vitro* and *in vivo* protein synthesis. Biochemistry.

[2] Shao HB, Liang ZS, Shao MA (2005). LEA proteins in higher plants: structure, function, gene expression and regulation. Colloids and Surfaces B: Biointerfaces.

[3] Hundertmark M, Hincha DK (2008). LEA (late embryogenesis abundant) proteins and their encoding genes in *Arabidopsis thaliana*. BMC Genomics.

[4] Liang Y (2016). Genome-wide identification, structural analysis and new insights into late embryogenesis abundant (*LEA*) gene family formation pattern in *Brassica napus*. Sci. Rep..

[5] Wang XS (2007). Genome-scale identification and analysis of *LEA* genes in rice (*Oryza sativa* L.). Plant Sci..

[6] Lan T, Gao J, Zeng QY (2013). Genome-wide analysis of the LEA (late embryogenesis abundant) protein gene family in *Populus trichocarpa*. Tree Genet. Genomes.

[7] Garay-Arroyo A, Colmenero-Flores JM, Garciarrubio A, Covarrubias AA (2000). Highly hydrophilic proteins in prokaryotes and eukaryotes are common during conditions of water deficit. J. Biol. Chem..

[8] Gal TZ, Glazer I, Koltai H (2004). An LEA group 3 family member is involved in survival of *C. elegans* during exposure to stress. FEBS Lett..

[9] Finn RD (2016). The Pfam protein families database: towards a more sustainable future. Nucleic Acids Res..

[10] Dure L (1989). Common amino acid sequence domains among the LEA proteins of higher plants. Plant Mol. Biol..

[11] Hunault G, Jaspard E (2010). LEAPdb: a database for the late embryogenesis abundant proteins. BMC Genomics.

[12] Battaglia M, Olvera-Carrillo Y, Garciarrubio A, Campos F, Covarrubias AA (2008). The enigmatic LEA proteins and other hydrophilins. Plant Physiol..

[13] Li X, Cao J (2016). Late embryogenesis abundant (*LEA*) gene family in maize: identification, evolution, and expression profiles. Plant Mol. Biol. Rep..

[14] Chen YK (2019). The role of the late embryogenesis-abundant (LEA) protein family in development and the abiotic stress response: a comprehensive expression analysis of potato (*Solanum Tuberosum*). Genes.

[15] Altunoglu YC, Baloglu P, Yer EN, Pekol S, Baloglu MC (2016). Identification and expression analysis of *LEA* gene family members in cucumber genome. Plant Growth Regul..

[16] Cao J, Li X (2015). Identification and phylogenetic analysis of late embryogenesis abundant proteins family in tomato (*Solanum lycopersicum*). Planta.

[17] Wu CL (2018). The late embryogenesis abundant protein family in Cassava (*Manihot esculenta Crantz*): genome-wide characterization and expression during abiotic stress. Molecules.

[18] Du DL (2013). Genome-wide identification and analysis of late embryogenesis abundant (LEA) genes in *Prunus mume*. Mol. Biol. Rep..

[19] Gao J, Lan T (2016). Functional characterization of the late embryogenesis abundant (LEA) protein gene family from *Pinus tabuliformis* (Pinaceae) in *Escherichia coli*. Sci. Rep..

[20] Battaglia M, Covarrubias AA (2013). Late Embryogenesis Abundant (LEA) proteins in legumes. Front. Plant Sci..

[21] Olvera-Carrillo Y, Reyes JL, Covarrubias AA (2011). Late embryogenesis abundant proteins: versatile players in the plant adaptation to water limiting environments. Plant Signal Behav..

[22] Bies-Ethève N (2008). Inventory, evolution and expression profiling diversity of the LEA (late embryogenesis abundant) protein gene family in *Arabidopsis thaliana*. Plant Mol. Biol..

[23] Manfre AJ, Lanni LM, Marcotte WR (2006). The arabidopsis group 1 late embryogenesis abundant protein ATEM6 is required for normal seed development. Plant Physiol..

[24] Huang Z (2016). Genome-wide identification, characterization, and stress-responsive expression profiling of genes encoding LEA (late embryogenesis abundant) proteins in Moso Bamboo (*Phyllostachys edulis*). PloS one.

[25] Muvunyi BP (2018). Mining *late embryogenesis abundant* (LEA) family genes in *Cleistogenes songorica*, a xerophyte perennial desert plant. Int. J. Mol. Sci..

[26] Tolleter D, Hincha DK, Macherel D (2010). A mitochondrial late embryogenesis abundant protein stabilizes model membranes in the dry state. Biochim. Biophys. Acta.

[27] Babu RC (2004). *HVA1,* a LEA gene from barley confers dehydration tolerance in transgenic rice (*Oryza sativa* L.) via cell membrane protection. Plant Sci..

[28] Hara M, Terashima S, Fukaya T, Kuboi T (2003). Enhancement of cold tolerance and inhibition of lipid peroxidation by citrus dehydrin in transgenic tobacco. Planta.

[29] Hara M, Fujinaga M, Kuboi T (2005). Metal binding by citrus dehydrin with histidine-rich domains. J. Exp. Bot..

[30] Krüger C, Berkowitz O, Stephan UW, Hell R (2002). A metal-binding member of the late embryogenesis abundant protein family transports iron in the phloem of *Ricinus communis* L. J. Biol. Chem..

[31] Hsing YC (1998). Tissue- and stage-specific expression of a soybean (*Glycine max* L.) seed-maturation, biotinylated protein. Plant Mol. Biol..

[32] Candat A (2014). The ubiquitous distribution of late embryogenesis abundant proteins across cell compartments in *Arabidopsis* offers tailored protection against abiotic stress. Plant cell.

[33] Upadhyaya H, Panda SK (2013). Abiotic stress responses in tea [*Camellia sinensis* L (O) Kuntze]: an overview. Rev. Agric. Sci..

[34] Chen Q, Yang LM, Ahmad P, Wan XC, Hu XY (2011). Proteomic profiling and redox status alteration of recalcitrant tea (*Camellia sinensis*) seed in response to desiccation. Planta.

[35] Paul A, Kumar S (2013). *Dehydrin2* is a stress-inducible, whereas *Dehydrin1* is constitutively expressed but up-regulated gene under varied cues in tea [*Camellia sinensis* (L.) O. Kuntze]. Mol. Biol. Rep..

[36] Paul A, Singh S, Sharma S, Kumar S (2014). A stress-responsive *late embryogenesis abundant protein 7 (CsLEA7)* of tea [*Camellia sinensis* (L.) O. Kuntze] encodes for a chaperone that imparts tolerance to Escherichia coli against stresses. Mol. Biol. Rep..

[37] Jin XF (2018). Transcriptome and expression profiling analysis of recalcitrant tea (*Camellia sinensis* L.) seeds sensitive to dehydration. Int. J. Genomics.

[38] Wang WD (2019). The late embryogenesis abundant gene family in tea plant (*Camellia sinensis*): Genome-wide characterization and expression analysis in response to cold and dehydration stress. Plant Physiol. Biochem..

[39] Xia EH (2017). The tea tree genome provides insights into tea flavor and independent evolution of caffeine biosynthesis. Mol. Plant.

[40] Wei CL (2018). Draft genome sequence of *Camellia sinensis* var. *sinensis* provides insights into the evolution of the tea genome and tea quality. Proc. Natl. Acad. Sci. USA.

[41] Marchler-Bauer A (2015). CDD: NCBI’s conserved domain database. Nucleic Acids Res..

[42] Gasteiger E (2003). ExPASy: the proteomics server for in-depth protein knowledge and analysis. Nucleic Acids Res..

[43] Horton P (2007). WoLF PSORT: protein localization predictor. Nucleic Acids Res..

[44] Larkin MA (2007). Clustal W and clustal X version 2.0. Bioinformatics.

[45] Kumar S, Stecher G, Tamura K (2016). MEGA7: molecular evolutionary genetics analysis version 7.0 for bigger datasets. Mol. Biol. Evol..

[46] Hu B (2015). GSDS 2.0: an upgraded gene feature visualization server. Bioinformatics.

[47] Bailey TL (2009). MEME Suite: tools for motif discovery and searching. Nucleic Acids Res..

[48] Livak KJ, Schmittgen TD (2001). Analysis of relative gene expression data using real-time quantitative PCR and the 2^−ΔΔCT^ method. Methods.

[49] Roach T (2010). Extracellular superoxide production, viability and redox poise in response to desiccation in recalcitrant *Castanea sativa* seeds. Plant Cell Environ..

[50] Magwanga RO (2018). Characterization of the late embryogenesis abundant (LEA) proteins family and their role in drought stress tolerance in upland cotton. BMC Genetics.

[51] Ling H, Zeng X, Guo SX (2016). Functional insights into the late embryogenesis abundant (LEA) protein family from *Dendrobium officinale* (Orchidaceae) using an *Escherichia coli system*. Sci. Rep..

[52] Nagaraju M (2019). Genome-scale identification, classification, and tissue specific expression analysis of late embryogenesis abundant (LEA) genes under abiotic stress conditions in *Sorghum bicolor* L. PloS one.

[53] Patil A, Nakamura H (2006). Disordered domains and high surface charge confer hubs with the ability to interact with multiple proteins in interaction networks. FEBS Lett..

[54] Fuxreiter M, Simon I, Friedrich P, Tompa P (2004). Preformed structural elements feature in partner recognition by intrinsically unstructured proteins. J. Mol. Biol..

[55] Pedrosa AM, Martins CPS, Gonçalves LP, Costa MGC (2015). Late embryogenesis abundant (LEA) constitutes a large and diverse family of proteins involved in development and abiotic stress responses in sweet orange (*Citrus sinensis* L. Osb.). PloS one.

[56] Chen Q (2019). Genome-wide identification of cyclophilin gene family in cotton and expression analysis of the fibre development in *Gossypium barbadense*. Int. J. Mol. Sci..

[57] Hou D (2018). The gibberellic acid-stimulated transcript gene family in Moso Bamboo:a genome-wide survey and expression profiling during development and abiotic stresses. J. Plant Growth Regul..

[58] Pan C (2017). Genome-wide analysis of the biosynthesis and deactivation of gibberellin-dioxygenases gene family in*Camellia sinensis* (L.) O. Kuntze. Genes.

[59] Wang YX (2018). Genome-wide identification and expression analysis of GRAS family transcription factors in tea plant (*Camellia sinensis*). Sci. Rep..

[60] Kalemba EM, Pukacka S (2012). Association of protective proteins with dehydration and desiccation of *orthodox* and *recalcitrant* category seeds of three *Acer* genus species. J. Plant Growth Regul..

[61] Delahaie J (2013). LEA polypeptide profiling of recalcitrant and orthodox legume seeds reveals ABI3-regulated LEA protein abundance linked to desiccation tolerance. J. Exp. Bot..

[62] Wang BF, Wang YC, Zhang DW, Li HY, Yang CP (2008). Verification of the resistance of a *LEA* gene from *Tamarix* expression in *Saccharomyces cerevisiae* to abiotic stresses. J. Forestry Res..

[63] Chiappetta A (2015). A dehydrin gene isolated from feral olive enhances drought tolerance in *Arabidopsis transgenic* plants. Front. Plant Sci..

[64] Wang MZ (2014). SiLEA14, a novel atypical LEA protein, confers abiotic stress resistance in foxtail millet. BMC Plant Bio..

