# Supplementary material for: Genome-wide identification and expression analyses of the LEA protein gene family in tea plant reveal their involvement in seed development and abiotic stress responses
Source: Sci Rep. 2019 Oct 1;9:14123. doi: 10.1038/s41598-019-50645-8 (PMC6773783; doi:10.1038/s41598-019-50645-8)
Supplement: Supplementary file 2 — Supplementary Table S1 [file 41598_2019_50645_MOESM2_ESM.docx]

**Genome-wide identification and expression analyses of the LEA protein gene family in tea plant reveal their involvement in seed development and abiotic stress responses**

**Xiaofang Jin^1, 2^, Dan Cao^1^, Zhongjie Wang^2^, Linlong Ma^1^, Kunhong Tian^2^, Yanli Liu^1^, Ziming Gong^1^, Xiangxiang Zhu^2^, Changjun Jiang^2,^ * & Yeyun Li^2,^ ***

^1^ Fruit and Tea Research Institute, Hubei Academy of Agricultural Sciences, Wuhan, 430064, China

^2^ State Key Laboratory of Tea Plant Biology and Utilization, Anhui Agricultural University, Hefei, 230036, China

* Correspondence: jiangcj@ahau.edu.cn; lyy@ahau.edu.cn

**Supplementary Table S1.** The amino acid sequences of the 48 CsLEA proteins and 51 AtLEA proteins.

| **Protein** | **Sequence** |
| --- | --- |
| *CsLEA1* | MSLAPPPPYAMMSDNNGSLRPPPYRRNIPRYHSHHKKGRNCCLKCICCFYCFLFLFLVIIAGSA  FYFYTFYKPNMPSYQVQGLTVQAFDLQPDFSLLTEFLVTVKAQNPNENVGFKYGRDNFVIVSY  KDSTLCTGHFPSFLQGHKNTTMINVLLKGKSEFGSGLQEALMQNRNSGKIPLLVQVKVPVNV  VVAEFSMSQFKVFVNCSLVVDNLSPNKKIGILSSKYNVGFSL |
| *CsLEA2* | MGNKVIWSWSSALVGAASAAAAAALISGKPRDPTFEVISISLTSFKLNLPVLDVELILTVHVTN  PNIVPISYSSSEMSIFYSGSKLGSALIDAGSQPPGSCRLLTLPARLSGLELAHHATQFIADVSRRE  MVLDAAVDIEGAAKVLWWDHKFKIHVDSHIIVDPVFLDVIDQENKSDLDVFVA |
| *CsLEA3* | MTSSTRPTFNTDHTSRLAPPPHHHRHYYPPPYSSSKASFKGCCCCLFLLFSFLALLILALFLVFF  FALKPKKPQFDLQQVGVQYLGISPTTPTSASVSLTIRLLFTAVNDNKVGIKYSESRFTVMYRGIP  LGRGVVPGFYQAAHSVREVETTVAVDRVSLLQADAAELVRDAELNDRVELRVLGDVGAKIRI  LGFTSPAVQVSVDCAIVISPRKQALTYKQCGFDGLTV |
| *CsLEA4* | MTTTKDEDPVVFYAPIPTNPDNSNQNYVVLPLYPHSHLRRRHNHHHHHRYPSLSRCVGVAAA  FLLLSTAAFLLYPSDPHLSLVRIHLNHIQILTSPQLSLDLSLSLTLRVLNRDFFSLDYSSVHVTIGY  RGRELGFVTSNGGHVKARGSSYVNATLVLDGFEVIHDVVYLIADLARGTIPFDTDSEVNGTVE  IFFIDVPIKPADPARKIGAQAHP |
| *CsLEA5* | MEVESTKDAKSIGARPENARNRRRNICLAVIAVVLVIVILLVVLGFTVFKAKHPINTVNSIAVKD  LKFSLDIMRLRVNLNVSLDVNLSIKNPNKVGFKYTNSSALLKYRGEVVGDVPVPAGEISAGET  LPMNMTLTVLADRLLSNSDVYSDVMSGTLPFSTFTRMSGKVHIMIFKIHLVSYTTCDLNISVG  NRSVTNQTCHYKTKL |
| *CsLEA6* | MSKPRHKPPSGRTNLASCIVATIFLIFVIIIILIVFFTVFKPRDPQISVNAVQLPTFSLANGTVNFTF  SQYVSVKNPNRAVFTHYDSTLQLLYSGSQIGFMFIPAGKIDAGRTQYMAATFSVQSFPMAAAA  APENVGPNVTDGLNGFRVGPTMEIESRMEMAGRVRLLHFFAHHVEAKSQCQVAISVSDGSVL  GFHC |
| *CsLEA7* | MARSFSNAKFLSAFVVDRISLAVTRRGYAAASQGVASGPVTARGGVMGKKGGEEEATKTSWV  PDPVTGYYRPENKANEIDVAELREMLLKNKIRRH |
| *CsLEA8* | MAEEQHHHHLGEEKSHECESTVISSTITDEVEVETQDRGLFGFMGKKEEEKKPEEEVIVTEFEK  VHVSEPEPKFQDCKEEEKKHGLLEKLHRSNSSSSSSSDEEEEDENGKKKKKKNKGPKEKIKGK  ISGEKEEEKIEKYEAVVTPLPPKEKYEEEVVVVHGELAQPEEKKGIFEKIKEKLPGQNKKAEED  PPPPPAAEYAAPAAETHSHEAEPKEKKGIFEKIKEKLPGYHPKTEEEKEKEKEKEKECASPTK |
| *CsLEA9* | MVRTGVTKTPMILLRAHKPAGRHTSELSKTDQVAEKHQGSIKGYKKANPSCWMPHPRTGIYF  PEGHDHVMDGVSESAASFDQTYWLRNVDGVDKPDHDECYFHPSL |
| *CsLEA10* | MARSLSNAKLLSAFIVDRISVSASRRGYAGASQGVAVSGSGGARAAMAKKGGEEVTKTSSWV  PDPKTGYYRPENHADDIDVAELRQMLLKNNIRRH |
| *CsLEA11* | MTSHYDSLATISGEERVGVNVGKKGKKEREKESHADKARRVNRVAEKEAEAESKKEKEEDQR  GGLSLEEIFKYRQSAQQNSMDAIRASEERYAKKAKELGSSALQNVKESAQQAKDYTAQTAHQ  ANDYASQKAVGAKEAGASVAEREPAKDVVVETGKSTVGYGGKTLLIVLCLVATRFEFGHIMG  EETGEQECGSGEVVEKPTETVQEELYWAAPEEHERREHQGGGGIFAAIVETIVEIAQNTKDLVV  GQDDQTRGYKGGSIEYTEPEEVEERRVP |
| *CsLEA12* | MHSAKEKVSNVVSEAKGKLDETKAKAEEKVEKATARTTEEERMAEEKRRAMEGEAKMGLH  EAKAEHAADKLQSGHHHPVGTAAPMAGATAPTYPLGGNPPGHHKYT |
| *CsLEA13* | MAEKEQQAYHPPANNGYSRNDEEAQAAEAEKLRLKKRKRLTFFVIFMVSQIVIGIVIGLTIMKV  KNPKFRVRSATFETFNVSNSSFNMTMNAQLGVKNPNFGPYKFRNTTVKFYYKETLVGTAIVSK  SKAGLKTTKKFDVTVDLIAPISLASDAQLASDISIGLLPLRSQSELSGKVELMLIMKKKKTTQM  NCTMNVNIKSQQLQDVMCK |
| *CsLEA14* | MASIQDNEERAEEAAVRVAVSEPSSSDANRERKRTYYQERAKMEEESATYTRDGKEAAKEKIS  SVADTAAKKARECKDSTLGKVIEYKDYATEKVREAKDSALEKVGEYKDYAAAKKAKDSALG  KAGDYKDYTVEKAKAVTMEKAREGKDSAVGKISELKESVVDAVKRAMDMLSGKNKEEAMV  SRL |
| *CsLEA15* | MAEKEEQAKPLSTTTNRSYQINGEHDHAISIDLKKHHHNPKYLKCCGYISALILILAFTILMLIF  TVFRVKEPVLNINSGKINGLDRASKNTIRLDTNLTVVADVSIENPNVVAFKFGNTTTTLYYGGV  VVGNGRNPAGVAEAGKTVRTNMTIDVMTEKILGVRRLTSDLSYGALGMRSYTSISGGVKILKI  IKKNVVVKVNCSMTFNITSQAIQDQICMQHMSF |
| *CsLEA16* | MADQSTPVTGYPAAGYRHPPPQSGNNTNGYPPAGASYPYAAPPPQAAYYYAQPYPDPRATFLR  RLFGILIASFIIAATIAFIVWLILRPRLPEFQVDSLSLSNFSLSSSSISANWDVGFTARNPNHKITLY  YDDISATVFCGEQRLSDTAIGPFVQGTRNETTVRATFAANSAYVTKEVVDGINSDRSSRGIVNF  NVEMKARVRYKAGAWRARQRFLRIFCQQLSVGVSKNSVGGTLIGGPRQCKVVM |
| *CsLEA17* | MAKVPVNTFLLLSRRAYTVAAESVVGVQRAVVRKAAVEYSSREAPAAREKEVFWMRDPKSG  NWIPETHFDEVDVAELREKLLPRKK |
| *CsLEA18* | MDNHQSSRPPVTGYPAPGYPPSNNPSGCATTAGTGFPYAAPPPRPAYYNPRYPYYPRATLFRRL  LAILIAFFIICGTIVFIIWLVLRPRFPDFRVDSLSLSNFNLSSSPSSLISAKWDVRFTVRNPNHKLSI  FYDHINAYVFYKSESLSDTTLPPFVQGTRNQTTLRASFATASSYVDDWAVEGINGERKSHGTVN  FNVRLLSRVRFKASWWRARHRILSVYCGDLTVGISSNGAGGTLVGGARECRVGI |
| *CsLEA19* | MSQVLTRSPKHCAKQGLKNIEEHYKKLFYAFSTFFLSILFLIFLVWLILHPSKPEFSLKEADLYQL  NLTAPLPHLLNSSIQFTLLSKNPNKKVGIYYDELQVYASYKGQQITVGTSLPPFYQDHDESNLL  AGSLTGTGLPAGPSFGYELGRDRAAGKLVMSLKMNGRLRWKVGTWVSGRNRFNANCVAVV  DFRTSGQLSSKQGTQCSTTV |
| *CsLEA20* | MANPRPYTAQPRQSKLVRTICMVLIALIVLIGLTVLIIWLSLKPKKPRYSIENASISNLNIANNHL  NGTFNFVISAFNPNSKVSIYYDKVEVYVFYDKDKLAASAGQPFFQPHRHETHLDEKLVAQNVT  LTGKVAKDLGLEKAAGNVELDVQMKAKVRFKYGSWKTGHRRLRVFCSPVFVQSSKKNSQTT  DCHVSL |
| *CsLEA21* | MADRVHPRDSPPSSGEASTTSSSAHRTDLPLKPSHPVPEKPVPPPATYVIQVPKDQIYRYPPPEN  ARRMQKLARRKPRRSCCCRCICWSLALLLLLIFLLAATAGVLYLIFRPQAPKYSVDSIAISGFNL  TSVSSISPEFDVTIRAQNPNKKIGIYYETGSSVSVYYSDVDLCNGALPAFYQPSKNLTVFQMAL  KGSSILLKSSVHSALVAQQKSGTVPFKLNLKAPVKIKVGAVKTWTITVKVKCDLTVDSLTPAA  KIVSKDCKYSVKLW |
| *CsLEA22* | MQAAENMALGNTQKGGVAAVMQSAAAVNERGGAVGHYDYTDVAREQGVTVTETVIGPRRI  ITKTVVDQATYI |
| *CsLEA23* | MTTRTNMNGHHHHHHHNHTSPPPPPPPQSQPPPPRPPHNHRNSHSNDHYYPPPYSSSSSSSASL  KGCCCCLFLLFSFLALLILAVVLVIVLAVKPKKPQFDLQQVGVQYVGISPNPPSTTATEPSSASV  SLNIRLLFTAANDNKVGIKYGESRFTVMYRGIPLGRGVVPGFYQAAHSVRQVETTVAVDRVNL  MQADAAELVRDATLNDRVELRVLGDVGAKIRILGITSPGVQVSTLPLYLTGNYSIYLGFGPGPS  GLVTRARGSPSPINNAERERVRCPPENRNNSGEEVPISEKKTLRFNGSLSVGHNYVNDIARFLW  KSIQFLPRSRKAGSQVVAREARDVVASFSDCLLACICFVSHLLVKLLANHSFVYHELSCSSCRC  AAMGLECGLNFSNANCERLVCCDGTEIRSQQIATDFKDSQICLFYWINSLISTENKGDVEAFHA  LQY |
| *CsLEA24* | MADNTKQPQLNGAYYGPSIPPPSKTSHRPGRSHGGCGCCGCLFALIFKILITLIFIAGIVVLVFWL  VFRPNKVKFHVTDATLTQFDLNTTTNTLYYDLSLNLTIRNPNKHIGIYYDRLDARVYYEDQRFA  AESLTPFYQGHKNTTELGFVFKSHNIVSLNSEIAKYDSEKSSGSYSIDMKLYLTVRFKLRSVKTP  KFKPKIECDLKVPLASNGSVSGSFETVRCGIDW |
| *CsLEA25* | MYELQPQPQPQPQPQPQPQPHYLRSRRSRSRTNVASCIVASVFLLFLAAVAAVVFFLLFKPKDPT  ISVDALQFPSFSVSNSTFNFTLFQFVSVTNPNRDDFTHYDSSLQLLYSGSQVGVVFIPAGQIGAG  RTQHMSAKFDVKEYPLGARSGQVSVAAASGGVVAPTMEIETRMKLVGRVRVLKVFTHRVESR  VRCGVVIEVSDGSVLGFHC |
| *CsLEA26* | MFSTIQIFGLLNLKHVHACSVIYLVERVSAHDPMQKDMAMQRKEDKINQAESNKQEAHEHNA  TARQSAAATTRVGGGTQSYTTGGGMGMENYSTSGPNMSAGYGYGTSHHGTSNMTEGGMVG  SQYPLGADIGAATENPVGGG |
| *CsLEA27* | MSQILTKSPKHCGNKQGLNVSKFYKKLFCTFSTIFLSILLLIFLVYFILHPSKPQFSLKETDIYELN  LSNTHLLNSSIQLTLLSKNPNNKVGIYYDVLQVYASYKGQQITTDTSLPPFYQGNQESNLLTAS  LVGVGLPVDPSFGYEVGRDQVAGKLVLNLKVNGRLRWKVGTWVSGRYRFNVNCVSIMDFGP  TIPTGPLSSKQGTECSTTV |
| *CsLEA28* | MSQEQPRRPQDVQQEPIKYGDVFAVSSDLAEKPIGLKDATKMQTAEATVFGETQKSGAAVVM  LAAAIVNERAGLVGSNEVADVTGDIGVTVTATDYGTRVITESIAGQVVGQYYESTQVQQQQQ  TTPGSLAQSKITIGEALEATAHIASNKTVDKRDTAAIQAAEVRATGSNVISPGGVAVAAQSEAS  LNAGLVRDEDKIKLTHVLTDWSGML |
| *CsLEA29* | MEDHQRIHPAHDSEAPQKPMALLMPSGSSKPDKGNPAEYPPFRCTIPVMYTKPPKRRSCCFQC  FCWTTSLLILLIVIITIVTAFIYVIFQPEIPKYSIDSMRITQFNLNNDTSLSAVLNVNFTATNPNKKI  GIYYEHGSHISMWYTDTQLCQGFLPKFYQSHQNVTVLDVALSGQTQNATDLLHSMQEQQQS  GSVPLNLRVKVPVRINLGTLKLMKWKFLVRCNLVVDSLATDNVIKIKSSRCKFRLRL |
| *CsLEA30* | MASSDKPEIVERDVKGKEHKEDDKDDGKGGFIDKVKDFIQDIGEKIEETIGFGKPTAEVAGIHF  PKINLEKADIVVDVLVKNPNPIPIPLIDINYLIESDGRKLISGLIPDAGTIHAHGSETVKIPVTLIYD  DIKNTYADIKPGSIIPYKVKVDLIVDVPVFGRLTLPIEKTGEIPIPYKPDIDVEKIHFERFSLEETVA  ILHLKLENKNDFDLGLTDLDYEVWLCDMSIGSADLAKSTKLDRNGISRIEIPITFRPKDFGSALW  DMIRGKGTGYSMKGNINVDTPFGAMKLPISKESGTTRLKKDADDDDDDEKMDK |
| *CsLEA31* | MADHQKIHPVHHDAEAPPPPPTAPLVPPGTSKSDNGDPAVEQYRPFRRTIPVIHSKPPKRRSCCC  KCFCWTTLLLFLIIIIIGIIAAIVYFVFQPKIPKYSVDSLQITQFSLNGVSLYATFNVNITATNPNKKI  GVYYENGSKITVLYTGTELCQGSLPVFYQGHRNTTVMDVALTGQTQNATVMLQSLQADQQA  GLGIPLDLRVRVPVRIKLGSLKLMKWKFLGRCNLVVDSLVTNNVVSIKSSSCKFKIRL |
| *CsLEA32* | MANLMEKAKNFVSEKVAEMKKPEASVMDVDLKDISRDAVTYNAKISVQNPYSHSVPICQISY  TLKSTGRVIASGTMPDPGSLMANDTTILDVALKVAHSVLVTLGKDIGADWDIDYELDLGLTLD  LPLIGNFTIPLSSKGEIKLPTLSDLWNK |
| *CsLEA33* | MTTKDCGHHHHDERRKFYRRLIAVILTFIILVLIVIFLIWIILRPTKPRFILLDATVYAFNISSPPNFL  TSNLQITLSSRNPNDRIGIYYDKLDVYASYRSQQITLPTLLPSTYQGHKDENVWSPFLYGNAIPV  APFLAESLAEDQVAGSVLINIRVDGRVRFKVGTFISGKYHLYVNCPAFISFGNRNNGIPVGPAIK  FQLVQNCHVDV |
| *CsLEA34* | MAEKELKAGHPPATNGYTRSDDEAQTAEAEKLRLKKRKRLTFFIIFMVSQIVIGIVIGLTIMKVK  NPKFRVRSATFETFNVTTSSFNMTMNAELGVKNPNFGPYKYRNTTVEFYYGVTLVGTAIVPKS  KASLLKTKKFNVAVNLIAPPSLLSDTKLASDISFGLLPLSSQSNLSGKVELMLIMKKKKSTQMS  CTMNVNIKTQQLQDVMCN |
| *CsLEA35* | MADKEQVRPLAPASIRPRSDDEETALYLKKAHRTKCIKCCGCIAALFVILVIVLIILIFTVFKVKD  PIIKMNSFKVNKLDLITGTTTPRPGSNMSLTADVSVKNPNVASFKYSNTTTTLFYRGTVIGEAR  GPPGQAKARRTMRMNVTVDIIMDRLTSNPNLQSDLNSSGLLSMTSFTRVGGRVKILKIIKKHV  IVKMNCTMFINLTSQGIQEMNCKRKVKL |
| *CsLEA36* | MVEGRSRGGQTRKEQLGTEGYQELGHRGGQTRKEQLGTEGYQEMGRKGGLSTTDKSGGER  AEEEGIEIDESKFRTTNP |
| *CsLEA37* | MKSEQESASSLRRKRNIKCLLYIVAGVILQTASTLVFVLTFMRIRNPKGRFGSVAVENLIVNSSTS  SPSFFMKLNAQVTVKNTNFGQFKFENSNATISYRGSHVGDFVITKACASARSTKKMNVTVTV  NSSADASNDLNLSSDISLGKLTLTSHATVSGKIHLFMVIKKKKSVKMNCTMDVDTMTKAIENL  YCK |
| *CsLEA38* | MTTKDCGHHHHDERRKFYLRLFAVILTIIILVLIVIFLIWIILRPTKPRFILLDATVYAFNISSPPNFL  TSNLQITLSSRNPNDRIGIYYDKLDVYAVYRRQQITLPTLLPASYQGHEDVSVWSPFLYGNAVP  VGPYVAAALCGDEAAGTVLINVKVDGRVRWKVGDFVSGKYHLYVKCAAYIPFGNKNSNSGI  VIGPGINKYQLVQNCHVDV |
| *CsLEA39* | MSGELASKPIAPQDAATMQAAENMALGNTQKGGVAAVMQSAAAVNERGGAVGHYDYTDVA  REQGVTVTETVIGPRRIITETVVDQVVGQYTQPGTGQGRSSGSIRSLGRQQASRSERCRRYTSG  RDESHWLQRDSLRWRSRHRAICSYSQRQDHA |
| *CsLEA40* | MSEKECSHHKDKRRKRLRRFFGGLLILIFIILIIVLLIWAILQPKKPRFVLQDATVYAFNVTAPNF  LTSTILVTIASRNPNDKIGVYYDKLDIFAIYQNQQITYYTAIPSTYQGHKEINVWSPIVSGVNVPV  APYNGVGLSQDQADGTVILTIKIQGRVRFKVGTFVSGHYHLSVNCPANIVFGNPTPGIVVGSNG  NPTPGIVVGSNGIKYQLDRSCSVSL |
| *CsLEA41* | MADIYDQFGNKIPLTDEHGHPVQLTDERGKPVHLTGVAVVEEAETGAGVVVTETVVSVGMTD  VFGYGHGAGAGAGSAGITDIGSTGGVGTAASDAHRQEQLHRSSSSSSSSSEDDGQGGRRKKK  KKKGLKEKIKEKLTGGKHKDDQHAHSATTATYPTNTTTTAGYPTTVSSTTPPGQQHHHEAVAA  GHEKKGIMEKIKEKLPGQHNH |
| *CsLEA42* | MTDRVYPSSKPTANGTTTTAGVANLSFPATKGQLYNAGRPPYRPLPPRHHRRQRSCCCSCCLW  TTLIVFLILLLSSIAGTIFWVLYRPHHPTFSLSSLQIPQFNLTPSSSSSKLSSKFNLTINAHNPNKKL  EFLYDPISVSISADGVDVGDGSIPGFVHFRKNTTILRTTIASNGQSMDSTSVSVLKSDLKNKKSL  PMKIELDTKVKVKIGGLKTNKVGVRVLCEGIKAAVPTGKKATMATTSNANCKVDLRIKIWKW  TF |
| *CsLEA43* | MSEKECSLHKHKKHKLLRRIFAGILITLFIILIIVLIIWAILQPKKPQFSLQDATVYSFNITAPNFLT  SAIQITISSRNPNDQIGVYYDKLDVYATYQNQQITYYTSIPPTYQGHMSVNVWSPFVSGTTVPV  APYNGVALSQDEADGTVNLLFKIDGRVRFKVGTFISAQYPLFVKCPAVITFGSRSTGVVVGDAV  KYQMFQSCSVTV |
| *CsLEA44* | MTEMEQIKPLAPSSHRIIFEDDHAISTELKKHRSLKFIKCCGCIVALVLILSVTLLVLIFTVLKVKE  PEMKMNSMMVQGLDRVNPTTNLTVIVDVSVKNPNVASFKFTNSTTSIYYGGMVVGEGRNPPG  IAKARRTLRMNVTIDVILEKVVGVERFSSDWSSRTLLMSSYTMVDGRVKIIGVFKKHVVVKM  NCTMTVNITSRGIQDQNCKHSVLL |
| *CsLEA45* | MAEREEQKAGHPLAPANGHARSDEEAQKEEAEELRLKKRKKRLIFFVIFTVSQIVMGIVFGLTI  MKVKTPKFRVRSATFETINVTSPSFHMKMYAEIGVKNPNFGPYKFHNTTVNFYYGETLFGSAN  IPKSIAGLKTTKKFNVTVDLIAPTSLASNLQFANELSSGLLPLRSQSNLSGKVELMLIMKKKKSI  QMNCTMDVNIKSQQLQDLNC |
| *CsLEA46* | MEYGQQGQHGHGTAGRVDQYGNPVGGVGHGTTGTGTGGMGTGYGTTGTGGMGQLGEHG  GAGMGGGQFQPVREEHNTGGILHRSGSSSSSSSEDDGMGGRRKKGIKEKIKEKLPGGRKDDQ  HATATGGTYGQQGHTGMTGTGTGGAYGTEGTGEKKGIMDKIKEKLPGQH |
| *CsLEA47* | MTTRTNMNGHHHHHHHNHTSRPPPPPPPQSQPPPPRPPHNHRNSHSNDHYYPPPYSSSSSSSAS  LKGCCCCLFLLFSFLALLILAVVLVIVLAVKPKKPQFDLQQVGVQYVGISPNPPSTTATEPSSAS  VSLNIRLLFTAANDNKVGIKYGESRFTVMYRGIPLGRGVVPGFYQAAHSVRQVETTVAVDRVN  LMQADAAELVRDATLNDRVELRVLGDVGAKIRILGITSPGVQVSIDCAIVISPKKQALTYKQCG  FDGLSV |
| *CsLEA48* | MAHNSNQYGNPPRQTDEYGNPPRKTDEFGDPVRQIDEYGNPVHHTGTMGDYGTTTGTTGVH  GTHTGTHTGTTGTYGTGTTGTGTTGTYGTGMDTTGTTGTHGLSTGTGGHHQQHADGGVLHR  SGSSSSSSEDDGQGGRRKKKGLTQKIKEKLPGGHKDQTPQYDNTTTTPGAATTGGYGYGGED  QQQYPEKKGMMEKIKEKLPGHTTTNK |
| At1g01470 | MASLLDKAKDFVADKLTAIPKPEGSVTDVDLKDVNRDSVEYLAKVSVTNPYSHSIPICEISFTF  HSAGREIGKGKIPDPGSLKAKDMTALDIPVVVPYSILFNLARDVGVDWDIDYELQIGLTIDLPV  VGEFTIPISSKGEIKLPTFKDFF |
| At1g02820 | MARSLANAKIQSVFGSEKLSNAVFRRGFAAAAKTALDGSVSTAEMKKRAGEASSEKAPWVPD  PKTGYYRPETVSEEIDPAELRAILLNNKQ |
| At1g03120 | MAQHQHSPQRPRDQDNTRPHDQYGIVFSVSGDDVARKQGDSFSQPDPTVATMGSVDTVTIGE  ALEATALSLGDKPVDRRDAAAIQAAETRATGESKGRPGGLAVAAQAAATTNEQTVSEEDKVN  IADILTDAAERLPGDKVVTSEDAEAVVGAELRSSSEMKTTPGGVADSMSAGARLNQQL |
| At1g20440 | MAEEYKNNVPEHETPTVATEESPATTTEVTDRGLFDFLGKKEEEVKPQETTTLESEFDHKAQIS  EPELAAEHEEVKENKITLLEELQEKTEEDEENKPSVIEKLHRSNSSSSSSSDEEGEEKKEKKKKI  VEGEEDKKGLVEKIKEKLPGHHDKTAEDDVPVSTTIPVPVSESVVEHDHPEEEKKGLVEKIKEK  LPGHHDEKAEDSPAVTSTPLVVTEHPVEPTTELPVEHPEEKKGILEKIKEKLPGYHAKTTEEEVK  KEKESDD |
| At1g20450 | MAEEYKNTVPEQETPKVATEESSAPEIKERGMFDFLKKKEEVKPQETTTLASEFEHKTQISEPES  FVAKHEEEEHKPTLLEQLHQKHEEEEENKPSLLDKLHRSNSSSSSSSDEEGEDGEKKKKEKKK  KIVEGDHVKTVEEENQGVMDRIKEKFPLGEKPGGDDVPVVTTMPAPHSVEDHKPEEEEKKGF  MDKIKEKLPGHSKKPEDSQVVNTTPLVETATPIADIPEEKKGFMDKIKEKLPGYHAKTTGEEEK  KEKVSD |
| At1g32560 | MQSAKQKISDMASTAKEKMVICQAKADEKAERAMARTKEEKEIAHQRRKAKEAEANMDMH  MAKAAHAEDKLMAKQSHYHVTDHGPHVPQQAPVPAPAPVMGHGYGHNPTGVTSVPPQTYH  PTYPPTGHHNHHHY |
| At1g52690 | MASHQEQSYKAGETRGKAQEKTGEAMGTMGDKTQAAKDKTQETAQSAQQKAHETAQSAKD  KTSQAAQTTQERAQESKDKTGSYMSETGEAIKNKAHDAAEYTKETAEAGKEKTSGILGQTGE  QVKQMAMGATDAVKHTLGLRTDEGNKEHVSSAPSTTTTTTTRETQRK |
| At1g54410 | MAGLINKIGDALHIGGGNKEGEHKKEEEHKKHVDEHKSGEHKEGIVDKIKDKIHGGEGKSHD  GEGKSHDGEKKKKKDKKEKKHHDDGHHSSSSDSDSD |
| At1g72100 | MTNLLALCLVLSTLLAAEVWSPSPAMTTHNTAVASEGEVIVKDGHHVVVVEYDRDGKTNTRV  SISPPSADQGEEKENEVEMGTSMFRNVKEKAKETASYLPHVGQGISQPVMTDEARDHHATAG  EVICDAFGKCRQKIASVVGRAKDRTVDSVGETASDVREAAAHKAHDVKETVTHAARDVEDT  VADQAQYAKGRVTEKAHDPKEGVAHKAHDAKESVADKAHDAKESVAQKAHDAKEKVREKA  HDVKETVAQKAHESKERAKDRVREKAQELKETATHKSKNAWERVKNGAREFGSATAATLSPT  KVASIVGLTGIAAAFGTSVWVTFVSSYVLASVLGRQQFGVVQSKLYPVYFKATSVGILVGLFGH  VLSRRRKLLTDATEMWQGVNLLSSFFMIEANKSFVEPRATKAMFERMKAEKEEGRGGERTSE  QELRRKLEQLSERLSKLNTYSSWLNILTLMSLTWHFVYLGQRLGAAC |
| At1g76180 | MAEEIKNVPEQEVPKVATEESSAEVTDRGLFDFLGKKKDETKPEETPIASEFEQKVHISEPEPEV  KHESLLEKLHRSDSSSSSSSEEEGSDGEKRKKKKEKKKPTTEVEVKEEEKKGFMEKLKEKLPG  HKKPEDGSAVAAAPVVVPPPVEEAHPVEKKGILEKIKEKLPGYHPKTTVEEEKKDKE |
| At2g03740 | MSISGAVLSGLGPSFLISGGKRSGVGGGAMKVGRKNVIIAPQRKKSWVSAAVKGAGNSPNDPK  WLDDASEKASGYVKEKGSEVGNVSAQKGQELQNQMERAKDYIFGKAGEAMDSVAENAKRA  SDFVTEKGKEVKEETTSRTDKAKDFIVEKAGDVKDTAMDMRNKTSKYVGDKATEAKEAILPP  KTDA |
| At2g03850 | MAMSISGAVFNGLGSSFLISRGKRSGVGLGVVAMRVGRKNVITSPKCKKSWVPTAVKGDGNSK  LDPKWLDDASQKASDYVKEKGSEVGHASAQKGQDVNDHVDRAKYYMFEKASEAIDNVAEI  AQFASEFVTEKGKETKKETASISEKAKDFIVEKAGEIIDIATDVSKKTAKYVGDKAKEVKEAIM  PPKT |
| At2g18340 | MMERRRTALVLFVVVVVLTWQEGVLGKWLESTAKEKTGSWAGWVSDKITTGFGTKKEETGI  YQKSKDEARKAAQAAENYAYDKANYVKDSAYDNAGYAKDFAENKAEYAKDFAYDKAGDAK  NMAYENAGYAKDFAYDKAGDAKDMAYEKAGHAKDFAYDKAGNAKDMAYETAGYAKDFAY  DKGGYAKDVAYDKAGNAKDMAYEKAGNAKDMAYEKAEHIKDFTYDKVGSAYGSAQSMMD  SGYDKAGDAKDMAYEKAGIVKDMAYDKAGDAKDVAYEKAGIAKDMAYDKAGNAKDMAYD  KVGSAYGSAQKAKDSGYEKAGEAKDYAYKKAGNAKDIAYEKAQDAKDFAYDKAGYGYDKA  GDVIRMATDKSGEAYEGAKEKSKSAKDTAGEAMDDSIDYMKDKSHNAKDGATRGFEEAMEK  VGEKYGVAKESTKYAYETAKKKASQVAGEIRDRYAEL |
| At2g21490 | MADLRDEKGNPIHLTDTQGNPIVDLTDEHGNPMYLTGVVSSTPQHKESTTSDIAEHPTSTVGET  HPAAAPAGAGAATAATATGVSAGTGATTTGQQHHGSLEEHLRRSGSSSSSSSEDDGQGGRRKK  SIKEKIKEKFGSGKHKDEQTPATATTTGPATTDQPHEKKGILEKIKDKLPGHHNHNHP |
| At2g23110 | MEDQKKPPTTEQEVKEVKNDDLESIKTPYLDYDNLEDYKMKGYGAQGHQEPKLGMGGGAT  DAPTPSGGLGRGGGAASTDLSSTDAINRQGVP |
| At2g23120 | MEAGKTPPTTTTTTEKKTEQVKDNDLPTDSPYMATGTLEDYKLKAYGAEGHQEPTPGLGGGS  TDAPTPSGDAPAATTTDAKAP |
| At2g33690 | MSKSEEKQELPLETSPYTKYEDIEDYKKNAYGTSGHQDVKPGHGGGTTDAPTPSGDAAPSAID  SANQKAKK |
| At2g35300 | MQSAKEKISDMASTAKEKLNIGGAKAQGHAEKTMARTKKEKKLAQEREKSKEAQAKADLHQ  SKAEHAADAQVHGHHLPGHSTYPTRATGANYPPGQI |
| At2g36640 | MASDKQKAERAEVAARLAAEDLHDINKSGGADVTMYKVTERTTEHPPEQDRPGVIGSVFRAV  QGTYEHARDAVVGKTHEAAESTKEGAQIASEKAVGAKDATVEKAKETADYTAEKVGEYKDY  TVDKAKEAKDTTAEKAKETANYTADKAVEAKDKTAEKIGEYKDYAVDKAVEAKDKTAEKAK  ETANYTADKAKEAKDKTAEKVGEYKDYTVDKAVEARDYTAEKAIEAKDKTAEKTGEYKDYT  VEKATEGKDVTVSKLGELKDSAVETAKRAMGFLSGKTEEAKGKAVETKDTAKENMEKAGEV  TRQKMEEMRLEGKELKEEAGAKAQEASQKTRESTESGAQKAEETKDSAAVRGNEAKGTIFGA  LGNVTEAIKSKLTMPSDIVEETRAAREHGGTGRTVVEVKVEDSKPGKVATSLKASDQMTGQTF  NDVGRMDDDARKDKGKL |
| At2g40170 | MASQQEKKQLDERAKKGETVVPGGTGGKSFEAQQHLAEGRSRGGQTRKEQLGTEGYQQMG  RKGGLSTGDKPGGEHAEEEGVEIDESKFRTKT |
| At2g41260 | MGNLKSLVLLALLFSFSVAVFANTSNDATHDEVKPSTEATHAIEAQKHDGKPQIAEAQVEANDP  VVEPQQDWGGRGGCRWGCCGGWWRGRCRYCCRSQAEASEVVETVEPNDVEPQQGGRGGG  GGGGGGRGGCRWGCCGGWWRGRCRYCCRSQAEASEVVETVEPNDVEPQQGGRGGGGGGG  GGRGGCRWGCCGGWWRGRCRYCCRSQAEANEVVETVEAQQAKP |
| At2g41280 | MGNLMSLVLVALLFSLSLAVIADTSNDATHVKEEVKPSTEATDAIEAEVEVNDAVVEPQQGLPG  GGCRFGCCGGYWWNGLCIYCCRSQAEANEVVKTVEPQKEEAKP |
| At2g42530 | MAMSLSGAVLSGMGSSFHNVGAKQSGVGTVRVGRKSELVVVAQRKKSLIYAVKSDGNILDDL  NEATKKASDFVTDKTKEALADGEKTKDYIVEKTIEANETATEEAKKALDYVTEKGKEAGNKA  AEFVEGKAEEAKNATKS |
| At2g42540 | MASSFHSGAKQSSFGAVRVGQKTQFVVVSQRKKSLIYAAKGDGNILDDLNEATKKASDFVTD  KTKEALADGEKAKDYVVEKNSETADTLGKEAEKAAAYVEEKGKEAANKAAEFAEGKAGEA  KDATK |
| At2g42560 | MASEQARRENKVTEREVQVEKDRVPKMTSHFESMAEKGKDSDTHRHQTEGGGTQFVSLSDK  GSNMPVSDEGEGETKMKRTQMPHSVGKFVTSSDSGTGKKKDEKEEHEKASLEDIHGYRANA  QQKSMDSIKAAEERYNKAKESLSHSGQEARGGRGEEMVGKGRDSGVRVSHVGAVGGGGGG  EEKESGVHGFHGEKARHAELLAAGGEEMREREGKESAGGVGGRSVKDTVAEKGQQAKESV  GEGAQKAGSATSEKAQRASEYATEKGKEAGNMTAEQAARAKDYALQKAVEAKETAAEKAQR  ASEYMKETGSTAAEQAARAKDYTLQKAVEAKDVAAEKAQRASEYMTETGKQAGNVAAQKG  QEAASMTAKAKDYTVQKAGEAAGYIKETTVEGGKGAAHYAGVAAEKAAAVGWTAAHFTTE  KVVQGTKAVAGTVEGAVGYAGHKAVEVGSKAVDLTKEKAAVAADTVVGYTARKKEEAQHRD  QEMHQGGEEEKQPGFVSGARRDFGEEYGEERGSEKDVYGYGAKGIPGEGRGDVGEAEYGRG  SEKDVFGYGPKGTVEEARRDVGEEYGGGRGSERYVEEEGVGAGGVLGAIGETIAEIAQTTKN  IVIGDAPVRTHEHGTTDPDYMRREHGQR |
| At2g44060 | MSTSEDKPEIISRVVHQEGDVEIVDRSQKDKDEEKEEGKGGFLDKVKDFIHDIGEKLEGTIGFG  KPTADVSAIHIPKINLERADIVVDVLVKNPNPVPIPLIDVNYLVESDGRKLVSGLIPDAGTLKAH  GEETVKIPLTLIYDDIKSTYNDINPGMIIPYRIKVDLIVDVPVLGRLTLPLEKCGEIPIPKKPDVDI  EKIKFQKFSLEETVAILHVRLQNMNDFDLGLNDLDCEVWLCDVSIGKAEIADSIKLDKNGSGLI  NVPMTFRPKDFGSALWDMIRGKGTGYTIKGNIDVDTPFGAMKLPIIKEGGETRLKKEDDDDD  DEE |
| At2g46140 | MASADEKVVEEKASVISSLLDKAKGFFAEKLANIPTPEATVDDVDFKGVTRDGVDYHAKVSV  KNPYSQSIPICQISYILKSATRTIASGTIPDPGSLVGSGTTVLDVPVKVAYSIAVSLMKDMCTDWD  IDYQLDIGLTFDIPVVGDITIPVSTQGEIKLPSLRDFF |
| At3g02480 | MDNKQNASYQAGQATGQTKEKAGGMMDKAKDAAASAQDSLQQTGQQMKEKAQGAADVV  KDKTGMNKSH |
| At3g15670 | MASNQQSYKAGETRGKAQEKTGQAMGTMRDKAEEGRDKTSQTAQTAQQKAHETAQSAKDK  TSQTAQAAQQKAHETAQSAKEKTSQTAQTAQQKAHETTQAAKEKTSQAGDKAREAKDKAGS  YLSETGEAIKNKAQDAAQYTKETAQGAAQYTKETAEAGRDKTGGFLSQTGEHVKQMAMGA  ADAVKHTFGMATEEEDKEHYPGSTTTTTATTRTTDPTHQTYQRK |
| At3g17520 | MGLERKVYGLVMVSLVLMAIATMCCVQATIEEEAAKDESWTDWAKEKIGLKHEDNIQPTHTT  TTVQDDAWRASQKAEDAKEAAKRKAEEAVGAAKEKAGSAYETAKSKVEEGLASVKDKASQ  SYDSAGQVKDDVSHKSKQVKDSLSGDENDESWTGWAKEKIGIKNEDINSPNLGETVSEKAKE  AKEAAKRKAGDAKEKLAETVETAKEKASDMTSAAKEKAEKLKEEAERESKSAKEKIKESYET  AKSKADETLESAKDKASQSYDSAARKSEEAKDTVSHKSKRVKESLTDDDAEL |
| At3g22490 | MSQEEQPKRPQEPVTYGDVFEVSGELADKPIAPEDANMMQAAETRVFGHTQKGGAAAVMQS  AATANKRGGFVHPGDTTDLAAERGVTVAQTDVPGARVTTEFVGGQVVGQYVEPRPVATAAA  MEAEVVGLSLQSAITIGEALEATVQTAGNKPVDQSDAAAIQAAEVRACGTNVIAPGGIAASAQ  SAANHNATIDRDEDKIKLIDVLAGATGKLAADKAVTRQDAEGVVSAELRNNPNLSTHPGGVAA  SITAAARLNERADI |
| At3g22500 | MSQEQPRRPREPVKYGDVFEVSGELADKPIAPEDAKMMQSAETHVFGHTQKGGPAAVMQSA  ATTNIRGGFVHPDDKTELVAERGATVEQTVPAATVTTEFVGGQVVGQHVEPRRVVAAARTDEE  ALQSTITIGEALEATVKTAGNKPVDQSDAAAIQAAEMRASGTNVIALAGVAASAQSAADHNAT  VDRDERKIKLRDVLTGAAGKLSADRAVTREDAEGVVSAEMRNNPKLCTHPGGVAASLTVAAR  LNERVDI |
| At3g50970 | MNSHQNQTGVQKKGITEKIMEKLPGHHGPTNTGVVHHEKKGMTEKVMEQLPGHHGATGTG  GVHHEKKGMTEKVMEQLPGHHGSHQTGTNTTYGTTNTGGVHHEKKSVTEKVMEKLPGHHG  SHQTGTNTAYGTNTNVVHHEKKGIAEKIKEQLPGHHGTHKTGTTTSYGNTGVVHHENKSTMD  KIKEKLPGGHH |
| At3g50980 | MESYQNQSGAQQTHQQLDQFGNPFPATTGAYGTAGGAPAVAEGGGLSGMLHRSGSSSSSSSED  DGLGGRRRKKKGITEKIKEKLPGHHDSNKTSSLGSTTTAYDTGTVHHEKKGMMEKIKEKLPG  GHH |
| At3g51810 | MASKQLSREELDEKAKQGETVVPGGTGGHSLEAQEHLAEGRSKGGQTRKEQLGHEGYQEIG  HKGGEARKEQLGHEGYQEMGHKGGEARKEQLGHEGYQEMGHKGGEARKEQLGHEGYKEM  GRKGGLSTMEKSGGERAEEEGIEIDESKFTNK |
| At3g53040 | MASGQREAERSAKAERAEAAASLAAEDLKDINKGDVTYKLTERTTTTEPERPGLIGSVMKAVQ  GTKDAVIGKSHDTAESTREGADIASEKAAGMRDTTGEVRDSTAQKTKETADYTADKAREAKD  KTADKTKETADYAAEKAREAKDRTADKTKETAEYTAEKAREAKDKTADKLGEYKDYTAEKA  KEAKDTTAEKLGEYKDYTVDKAKEAKDKTAEKAKETAEYTSDKARETKDKTAEKVGEYKDY  TAEKAKETADKAREAKDKTAEKVGEYRDYTAEKATETKDAGVSKIGELKDSAVDTAKRAMGF  LSGKTEETKQKAVETKDTAKEKMDEAGEEARRKMEEMRLEGKKLDEDASRKTQQSTESAAD  KAHETKDSVAQRGEEGKGSIMGALGNMTGAIKSKLTGATTPSDEETRASAHGDESTGKTVVAV  DVKDTRPGYVATVLKEADQMTGQTFNDVGEIDDEEKVRIVVGEKKL |
| At3g53770 | MSQSLFNLKSLSRSINNTIRMRRYIVITKASQRAYTIGSSQEKPSWASDPDTGYFRPETAAKELD  PYIAKTSQVQGKMMRGEELWWMPDPQTGYYRPDNFARELDAVELRSLHFNKNQKTYVVS |
| At4g02380 | MARSISNVKIVSAFVSRELSNAIFRRGYAATAAQGSVSSGGRSGAVASAVMKKKGVEESTQKIS  WVPDPKTGYYRPETGSNEIDAAELRAALLNNKQ |
| At4g13230 | MTSFAVVARLITRAPRVRASVPTRLVHGTTSTRKDSVCDKATEAQQKVAKKADEGAQTISDAA  GNLKDKAKNTAEEAWDKVKDTTEKIKDTVTGKTEETKESIKATAKTVERSMNTKNLK |
| At4g13560 | MSQQQFNAGQNRGQAQEKAEQWTESAKQTAQSACDKTADLTQSARDKAADLTQSARDKTA  DGSHSANKSAQHNQEQAAGLFGQTGESVKNMAQGALDGVKNSLGMNEKK |
| At4g15910 | MAARSLSGAVKSLCSAASGSLSCSIVLRRSYVATSQNVTAAGLSKGGSTRVMVGKMEQRGLD  QEAESAWGPDPVTGYYRPSNRAAEIDPAELRELLLKNKAKSF |
| At4g21020 | MAAMQLTRTALVGLSKAFPGIKAPATLAASSRKVSRICFATSVSQNEGRDPLDNARDSRADSAY  GSKKWREDTGEYYAQAAKDKANEGASKAADKAYETKEQAKDKAYETKEKAKDTAYNAKEK  AKDYAERTKDKVNEGAYKAADKAEDTKEKAKDYAEDTMDNAKEKARHAKEKVKEYGEDT  KEKAEGFKETVKGKAEELGEKTKETVKGAWESTKNAAQTVTEAVVGPEEDAEKARADMNK  GVEDHRKKKAEKDQKEDDFITFN |
| At4g36600 | MMIMMLTTVVTLTWQKQCYGWGTETAEDMVRNEAEHAKNAAETAKKMASDAAHDTKDKT  ASWAGWVSDKISTGLGGKKAEAEEAAESAKNYAYDKAGSAYDNAGYAKDFASDKAGSAYDS  AHNAKHYAYDKAGDAKDMAYDKTGQAKYMAYDKAGSAYEKAGQAKDMAYDKAGQAKD  MAYDKVGSAYDKAGQAKDMAYDKAGSASEKAGQAKDFAYDKAAHAKDAAYNKAEDVIKM  ATDTSGEAKDSAYGTYERFKEGSKNAKDIASDKAHDVRETAGRAVDYAKDKANDAYESGSEA  AGRFDEAMHKVGERYGAAKDSMSENTKEAYESAKEKASDAAGEYGSYMRDRSAEL |
| At4g38410 | MADHPRSTEQQEADAAASKGCGMFDFLKKKPEDVHSSENARVTKEPKEEEKPSLAERFHLSD  SSSSDEEAGENGEKKEKKKKKKKNEVAEDQCETEEKIPAGIGHEDGKEKGFMEKIKDKLPGGH  NGKPEAEPHNDKAKEKGFMEKIKEKLPGHTNDEKKKET |
| At4g39130 | MADLKDERGNPIYLTDAHGEPAQLMDEFGNAMHLTGVATTVPHLKESSYTGPHPITAPVTTTN  TPHHAQPISVSHDPLQDHDLRWFGTSSTEENGEGVGRKTNITDETKSKLGVDKPSAATVTGSG  SGSVHEKKGFFKKIKEKLSGHHNDL |
| At5g06760 | MQSMKETASNIAASAKSGMDKTKATLEEKAEKMKTRDPVQKQMATQVKEDKINQAEMQKR  ETRQHNAAMKEAAGAGTGLGLGTATHSTTGQVGHGTGTHQMSALPGHGTGQLTDRVVEGTA  VTDPIGRNTGTGRTTAHNTHVGGGGATGYGTGGGYTG |
| At5g27980 | MSEEQLQKPIDCADVKGEAEKISTTEGGIKAAEDKEKGVVAEASGEQAEGEVNQKKVVANPL  KSEGTITIGEALEAAVLTAGNKPVEWSDAAAIQAAEVRATGRTNIMPGGVAASAQSAATLNARI  GSDDTKTTLADVLTGASSKLPSDKAATRKDAEGVTGAEMRNDPHLTTYPTGVAASVAAAARI  NQSK |
| At5g44310 | MCEEISMVVDFQGRYDPVEKARDSRADLAYDSKKWREESGEYAEAGKGKAHKTKEEAKDKA  YDMKERTKDYAEQTKNKVNEGASRAADKAYETKEKAKDKAYDVKEKTKDYAEEAKDKVNE  GASRAADKAYETKEKAKDKAYDVKEKTKDFAEETKEKVNEGASRAADKAYDVKEKTKNYA  EQTKDKVNEGASRAADKAEETKDKAKDYAEDSKEKAEDMAHGFKEKAQDIGEKTMDTVKD  VWETAKSTAQKVTEAVVGSGEEADKARDDVDKGLEDLSKKAKENRNKDDDFKRF |
| At5g53260 | MGSSKDSASVTNISVEEHFSVSQSSPGGQFVGPTEEISTAAEALIGRSTTLTEALKAASMNVGH  KPVETTDVAAIKEVETRAIGGDIESEGGVTAVASKAVARNQKIGKDNEKTNLGDVIAEIDVKVT  RDREVTSEDAEAVIRAELNHSPFNNIIPGGVAESVAAAYKLNHDPSSL |
| At5g53270 | MMFGFGLLKQYAGTTEQISTAAEALVGRSTTLTEALKAAAINVGRKPVETTDLAAIKEVEARAI  GGDIESDGGVTAVASKAVARNQKIGEDNEKTNLGDVIAEIDVKVTRDREVTSEDAEAVIRAELN  HSPFNNIIPGGVAESVTAAYKLNCNPCNVSL |
| At5g66400 | MASYQNRPGGQATDEYGNPIQQQYDEYGNPMGGGGYGTGGGGGATGGQGYGTGGQGYGSG  GQGYGTGGQGYGTGTGTEGFGTGGGARHHGQEQLHKESGGGLGGMLHRSGSGSSSSSEDDG  QGGRRKKGITQKIKEKLPGHHDQSGQAQAMGGMGSGYDAGGYGGEHHEKKGMMDKIKEKL  PGGGR |
