# Supplementary material for: Genome-wide identification and expression analyses of the LEA protein gene family in tea plant reveal their involvement in seed development and abiotic stress responses
Source: Sci Rep. 2019 Oct 1;9:14123. doi: 10.1038/s41598-019-50645-8 (PMC6773783; doi:10.1038/s41598-019-50645-8)
Supplement: Supplementary file 3 — Supplementary Table S2 [file 41598_2019_50645_MOESM3_ESM.docx]

**Genome-wide identification and expression analyses of the LEA protein gene family in tea plant reveal their involvement in seed development and abiotic stress responses**

**Xiaofang Jin^1, 2^, Dan Cao^1^, Zhongjie Wang^2^, Linlong Ma^1^, Kunhong Tian^2^, Yanli Liu^1^, Ziming Gong^1^, Xiangxiang Zhu^2^, Changjun Jiang^2,^ * & Yeyun Li^2,^ ***

^1^ Fruit and Tea Research Institute, Hubei Academy of Agricultural Sciences, Wuhan, 430064, China

^2^ State Key Laboratory of Tea Plant Biology and Utilization, Anhui Agricultural University, Hefei, 230036, China

* Correspondence: jiangcj@ahau.edu.cn; lyy@ahau.edu.cn

**Supplementary Table S2.** Primers used for qRT-PCR analysis of *CsLEA* genes of *C. sisnesis*.

| **Gene name** | **Forward primer** | **Reverse primer** | **Amplification length (aa)** |
| --- | --- | --- | --- |
| *GAPDH* | TTGGCATCGTTGAGGGTCT | CAGTGGGAACACGGAAAGC | 206 |
| *CsLEA1* | GTGAGTTCGGGTCGGGTCT | GGCCACCACAACATTGACAG | 104 |
| *CsLEA2* | GGCAAACCGAGAGACCCAA | AGGAGATGGGGACGATGTTG | 133 |
| *CsLEA3* | TCCCAGCCAACTACCATAACC | GCTCTCCACCTTCTTCCTCTTG | 114 |
| *CsLEA4* | TATGTGAACGCCACGCTTGT | GAAGAAGATCTCAACCGTGCC | 135 |
| *CsLEA5* | ACCAACTCCTCGGCTTTGTT | CGTCAGCGTCATATTCATCG | 111 |
| *CsLEA6* | CCGGAGGAGGGAGAGTAATG | ACCACCCCATAGACCAGGAG | 121 |
| *CsLEA7* | ACAGAAGGACCCTTCGTCCAT | TTAAGGATCCACCGAGAGCAC | 106 |
| *CsLEA8* | AGTAAAGCCACGTGCACACTC | CACTAACCCGCGTTATTTTCC | 84 |
| *CsLEA9* | CATCCTCGCACCGGAATATAC | CATCAACGTTCCTCAGCCAA | 109 |
| *CsLEA10* | TCAATCTAAGAAGGGCCAAGC | ACCAAACCTCTCCCAATCCTC | 109 |
| *CsLEA11* | GTTGGGATCTTCGGCCTTG | CTCTCCGCTACAGAAGCACCT | 144 |
| *CsLEA12* | AACGGCGAGGACAACAGAA | CTCTGCCTTGGCTTCATGC | 97 |
| *CsLEA13* | CCGAGGGGGATGGTATCTG | ATGAAACTATGCGTGAAGCATCA | 84 |
| *CsLEA14* | GAGGGTAGCTGTTTCTGAGCCT | GTTGCCGACTCTTCCTCCAT | 99 |
| *CsLEA15* | AGATCGTGCCAGCAAGAACA | CACCACACCGCCGTAGTAAA | 136 |
| *CsLEA16* | AAATATGAGGTGGCGGGAATC | AAGGGAACCGAAAGAGTTTGG | 150 |
| *CsLEA17* | AGAGGGCAGTGGTGAGGAAG | TGAGCTCGGCAACATCAACT | 150 |
| *CsLEA18* | GGATCCTCTGTTCCTTGTTCTG | CCAAAACGCTCGTGCATTAA | 102 |
| *CsLEA19* | TCGGCAAATCACTTATGGGAG | AGTCACGGGTTCAATTTCCCT | 104 |
| *CsLEA20* | GTGATAAGCGCATTCAATCCC | GCCGCCAACTTGTCTTTGTC | 92 |
| *CsLEA21* | CGTTTTACCAGCCGTCAAAG | AAACGGCACCGTTCCACT | 125 |
| *CsLEA22* | CGCTCGGAAACACACAGAAG | CGTTACGGTGACTCCCTGTTC | 131 |
| *CsLEA23* | GAGAGTGAGATGTCCGCCTG | TCCATAGAAACCGAGCAATGTC | 137 |
| *CsLEA24* | GCCTCTTCGCCCTCATCTT | GTCGGTGACGTGGAATTTGA | 116 |
| *CsLEA25* | TGTTGGCTTTGTTTTCTGGC | AACCCGTCATCACCACCAC | 94 |
| *CsLEA26* | GTGGTGGGACCCAAAGTTACA | GGGTACTGTGACCCGACCAT | 154 |
| *CsLEA27* | CGGCATTTACTACGACGTGCT | ACTCCAACCAAAGACGCTGTT | 132 |
| *CsLEA28* | TCGCTGGACAGGTTGTAGGA | CCGTGGCTTCAAGTGCTTC | 120 |
| *CsLEA29* | CCCAACAAGAAGATCGGCAT | TGACAAAGCCACGTCGAGC | 141 |
| *CsLEA30* | TGACTTTGATTTGGGCCTCAC | GATGGGAATTTCAATGCGACT | 130 |
| *CsLEA31* | TCACCGGAACACGACAGTG | CAAATCCAGCGGAATCCCTA | 118 |
| *CsLEA32* | GGTGGCACATAGCGTATTGG | GATGAGCGGAAGATCAAGGG | 103 |
| *CsLEA33* | TGTCCTGAAAGTGGAGAGCAAG | TGCTGTAAGCTTCACCTTCACAA | 128 |
| *CsLEA34* | ATCATGCCGGCTTTTTCATC | GGCCCATCAATGTACGGTC | 182 |
| *CsLEA35* | TCTATCCGCCCTCGTAGTGAT | AAAGAGAGCAGCTATGCAACCA | 102 |
| *CsLEA36* | ATCAGGAACTGGGACACCG | TGGACTCGTCAATCTCGATCC | 153 |
| *CsLEA37* | CCGGAAGCGAAACATCAAG | TCGAATGCGCATGAAAGTG | 100 |
| *CsLEA38* | TTTGCCATAACTTCCCTGCC | AAACAATTCCCACTTTGTGAACTG | 87 |
| *CsLEA39* | CGCATCATCACTGAGACCGT | TTGTTGCCTGCCGAGAGAG | 108 |
| *CsLEA40* | TGTAGTGGAGGGGAGGTTGTG | CAAAGCTGTACTCATTTCCCCAAT | 94 |
| *CsLEA41* | GCTCGTCCGAGGATGATG | GTGGCAGTAGTGGCGCTATG | 136 |
| *CsLEA42* | CAACAATCGCATCGAATGGT | TCAGCCCTCCAATCTTCACTT | 135 |
| *CsLEA43* | CGTTAATGTATTGCTACTCTCTCA | TTCTCCTCTTCGTATCCATCAGC | 133 |
| *CsLEA44* | GGGCATTGCTAAGGCTAGGA | TGTTCTCGAACTCCAATCGCT | 109 |
| *CsLEA45* | CTCCCAAGTTTCGGGTTAGGT | ATTCTTGACGCCAATTTCAGC | 98 |
| *CsLEA46* | CAGCTCGTCCGAGGATGA | TCCCTGCTGCCCATAGGT | 136 |
| *CsLEA47* | CTCCCACTCCAACGACCACT | AGAAGAGGAGGAAGAGGCAGC | 101 |
| *CsLEA48* | TACCGGAGGCCATCATCAG | GTTGTCGTACTGCGGTGTCTG | 178 |
