# Supplementary material for: Genome-wide identification and expression analyses of the LEA protein gene family in tea plant reveal their involvement in seed development and abiotic stress responses
Source: Sci Rep. 2019 Oct 1;9:14123. doi: 10.1038/s41598-019-50645-8 (PMC6773783; doi:10.1038/s41598-019-50645-8)
Supplement: Supplementary file 4 — Supplementary Table S3 [file 41598_2019_50645_MOESM4_ESM.docx]

**Genome-wide identification and expression analyses of the LEA protein gene family in tea plant reveal their involvement in seed development and abiotic stress responses**

**Xiaofang Jin^1, 2^, Dan Cao^1^, Zhongjie Wang^2^, Linlong Ma^1^, Kunhong Tian^2^, Yanli Liu^1^, Ziming Gong^1^, Xiangxiang Zhu^2^, Changjun Jiang^2,^ * & Yeyun Li^2,^ ***

^1^ Fruit and Tea Research Institute, Hubei Academy of Agricultural Sciences, Wuhan, 430064, China

^2^ State Key Laboratory of Tea Plant Biology and Utilization, Anhui Agricultural University, Hefei, 230036, China

* Correspondence: jiangcj@ahau.edu.cn; lyy@ahau.edu.cn

**Supplementary Table S3.** The conserved motifs of different groups in CsLEA proteins.

| **Group** | **Motif** | **E-value** | **Consensus sequence** |
| --- | --- | --- | --- |
| LEA_1 | 1 | 5.1e-008 | 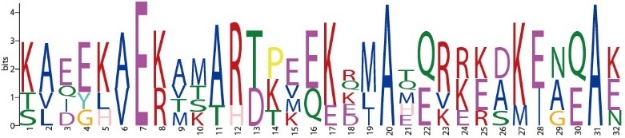 |
|  | 3 | 9.8e+000 | 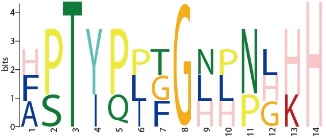 |
| LEA_2 | 1 | 3.2e-116 | 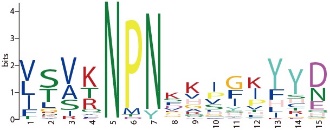 |
|  | 2 | 1.3e-157 | 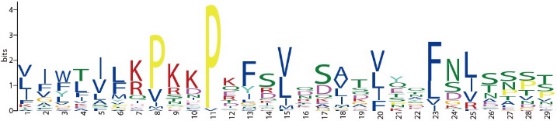 |
| LEA_3 | 1 | 4.9e-093 | 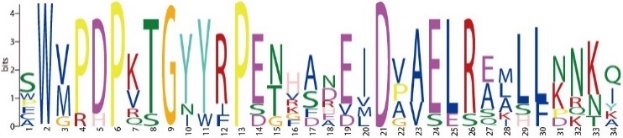 |
| LEA_4 | 1 | 8.7e-051 | 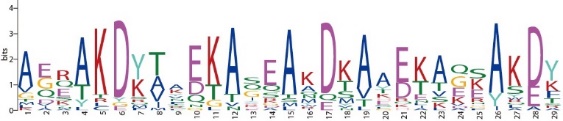 |
|  | 2 | 9.5e-034 | 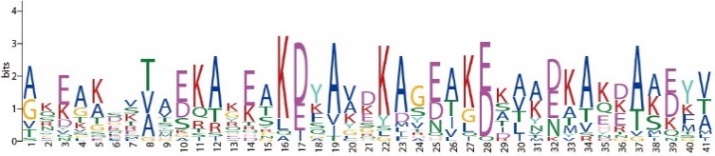 |
| LEA_5 | 1 | 4.9e-019 | 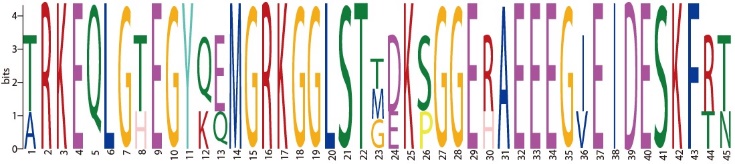 |
|  | 3 | 5.1e+001 | 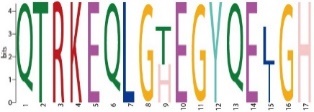 |
| SMP | 1 | 8.3e-061 | 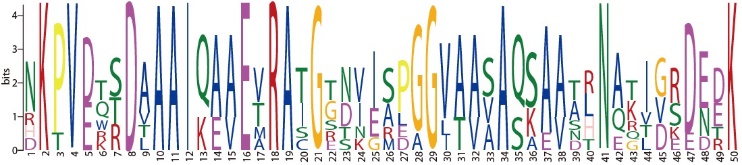 |
|  | 3 | 4.3e-049 | 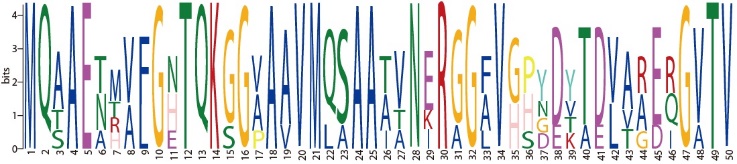 |
| DHN | 1 | 1.2e-082 | 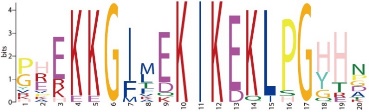 |
|  | 2 | 1.3e-071 | 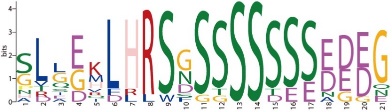 |
|  | 3 | 6.4e-052 | 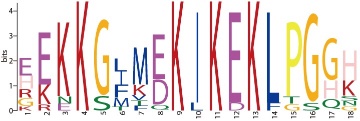 |
|  | 4 | 6.5e-023 | 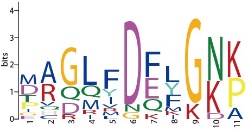 |
