# Supplementary material for: Genome-wide identification and expression analyses of the LEA protein gene family in tea plant reveal their involvement in seed development and abiotic stress responses
Source: Sci Rep. 2019 Oct 1;9:14123. doi: 10.1038/s41598-019-50645-8 (PMC6773783; doi:10.1038/s41598-019-50645-8)
Supplement: Supplementary file 5 — Supplementary Table S4 [file 41598_2019_50645_MOESM5_ESM.docx]

**Genome-wide identification and expression analyses of the LEA protein gene family in tea plant reveal their involvement in seed development and abiotic stress responses**

**Xiaofang Jin^1, 2^, Dan Cao^1^, Zhongjie Wang^2^, Linlong Ma^1^, Kunhong Tian^2^, Yanli Liu^1^, Ziming Gong^1^, Xiangxiang Zhu^2^, Changjun Jiang^2,^ * & Yeyun Li^2,^ ***

^1^ Fruit and Tea Research Institute, Hubei Academy of Agricultural Sciences, Wuhan, 430064, China

^2^ State Key Laboratory of Tea Plant Biology and Utilization, Anhui Agricultural University, Hefei, 230036, China

* Correspondence: jiangcj@ahau.edu.cn; lyy@ahau.edu.cn

**Supplementary Table S4.** The expression levels of 48 *CsLEA* genes in the roots, stems, leaves, flowers and seeds of tea plant.

| **Gene name** | **Values (Mean ± SD)** | | | | |
| --- | --- | --- | --- | --- | --- |
|  | **Root** | **Stem** | **Leaf** | **Flower** | **Seed** |
| *CsLEA1* | 0.00425±0.00051 | 0.00008±0.00002 | 0.00025±0.00001 | 0.03551±0.00484 | 0.00188±0.00009 |
| *CsLEA2* | 0.01072±0.00072 | 0.00052±0.00001 | 0.00852±0.00150 | 0.00045±0.00005 | 0.04266±0.00511 |
| *CsLEA3* | 0.00415±0.00056 | 0.00020±0.00006 | 0.00077±0.00001 | 0.00015±0.00004 | 0.00250±0.00085 |
| *CsLEA4* | 0.02874±0.00218 | 0.02077±0.00206 | 0.02188±0.00094 | 0.01939±0.00092 | 0.03141±0.00956 |
| *CsLEA5* | 0.01110±0.00236 | 0.03847±0.00589 | 0.02563±0.00146 | 0.16581±0.02387 | 0.04943±0.00085 |
| *CsLEA6* | 0.00709±0.00058 | 0.00020±0.00005 | 0.00034±0.00000 | 0.00038±0.00005 | 0.00338±0.00013 |
| *CsLEA7* | 0.00781±0.00086 | 0.00019±0.00005 | 0.00065±0.00002 | 0.00046±0.00005 | 0.00242±0.00021 |
| *CsLEA8* | 0.01625±0.00048 | 0.00016±0.00006 | 0.00064±0.00009 | 0.00051±0.00012 | 0.00371±0.00012 |
| *CsLEA9* | 0.00726±0.00063 | 0.05840±0.00232 | 0.00030±0.00004 | 0.00871±0.00085 | 0.00239±0.00045 |
| *CsLEA10* | 0.13107±0.00402 | 0.00077±0.00011 | 0.00421±0.00083 | 0.00294±0.00048 | 0.04520±0.00400 |
| *CsLEA11* | 0.00427±0.00089 | 0.00023±0.00002 | 0.00049±0.00004 | 0.00027±0.00006 | 0.26871±0.01894 |
| *CsLEA12* | 0.00672±0.00098 | 0.00010±0.00003 | 0.00045±0.00009 | 0.00033±0.00005 | 2.52198±0.51600 |
| *CsLEA13* | 0.08350±0.00674 | 0.00292±0.00070 | 0.00099±0.00011 | 0.00350±0.00022 | 0.00116±0.00005 |
| *CsLEA14* | 0.02510±0.00907 | 0.00029±0.00008 | 0.00136±0.00041 | 0.00121±0.00040 | 0.13379±0.02444 |
| *CsLEA15* | 0.32877±0.00837 | 0.00079±0.00017 | 0.00138±0.00010 | 0.00142±0.00024 | 0.00140±0.00050 |
| *CsLEA16* | 0.00808±0.00098 | 0.00009±0.00002 | 0.00058±0.00012 | 0.00056±0.00013 | 0.00204±0.00071 |
| *CsLEA17* | 0.15593±0.00339 | 0.00135±0.00014 | 0.02033±0.00149 | 0.00602±0.00015 | 0.00853±0.00125 |
| *CsLEA18* | 0.02837±0.00507 | 0.01514±0.00149 | 0.02434±0.00305 | 0.05838±0.00211 | 0.12914±0.02243 |
| *CsLEA19* | 0.00291±0.00015 | 0.00006±0.00001 | 0.00020±0.00007 | 0.00017±0.00006 | 0.00152±0.00048 |
| *CsLEA20* | 0.01620±0.00055 | 0.04827±0.00374 | 0.00296±0.00072 | 0.00261±0.00018 | 0.00347±0.00036 |
| *CsLEA21* | 0.26028±0.05108 | 0.00695±0.00038 | 0.00907±0.00068 | 0.08547±0.00085 | 0.01449±0.00245 |
| *CsLEA22* | 0.00041±0.00005 | 0.00001±0.00000 | 0.00002±0.00000 | 0.00002±0.00000 | 0.00884±0.00091 |
| *CsLEA23* | 0.00070±0.00007 | 0.00006±0.00000 | 0.00014±0.00001 | 0.00016±0.00005 | 0.00046±0.00012 |
| *CsLEA24* | 0.53683±0.07890 | 0.01178±0.00188 | 0.03612±0.00719 | 0.12548±0.01166 | 0.09547±0.00255 |
| *CsLEA25* | 0.00473±0.00042 | 0.01167±0.00069 | 0.00524±0.00062 | 0.00151±0.00021 | 0.02672±0.00041 |
| *CsLEA26* | 0.06513±0.00494 | 0.00249±0.00061 | 0.00614±0.00065 | 0.00822±0.00034 | 0.02520±0.00090 |
| *CsLEA27* | 0.02098±0.00336 | 0.02661±0.00616 | 0.06889±0.00335 | 0.01102±0.00037 | 0.00339±0.00037 |
| *CsLEA28* | 0.00174±0.00029 | 0.00005±0.00001 | 0.00009±0.00000 | 0.00704±0.00017 | 0.45007±0.07074 |
| *CsLEA29* | 0.03305±0.00982 | 0.00272±0.00062 | 0.00978±0.00196 | 0.00145±0.00011 | 0.00630±0.00153 |
| *CsLEA30* | 0.43387±0.02435 | 0.32810±0.03249 | 0.12324±0.00667 | 0.56062±0.04921 | 0.33120±0.00771 |
| *CsLEA31* | 0.26631±0.02192 | 0.00081±0.00009 | 0.00100±0.00007 | 0.00529±0.00001 | 0.00106±0.00013 |
| *CsLEA32* | 0.09933±0.00590 | 0.13315±0.02014 | 0.09810±0.00656 | 0.33879±0.02932 | 1.25786±0.11744 |
| *CsLEA33* | 0.00536±0.00069 | 0.00050±0.00012 | 0.00046±0.00015 | 0.00997±0.00164 | 0.00985±0.00082 |
| *CsLEA34* | 0.04059±0.00605 | 0.00013±0.00003 | 0.00053±0.00009 | 0.00042±0.00008 | 0.00383±0.00085 |
| *CsLEA35* | 0.02318±0.00050 | 0.00021±0.00000 | 0.00785±0.00151 | 0.00038±0.00002 | 0.00196±0.00004 |
| *CsLEA36* | 0.04694±0.00264 | 0.00113±0.00005 | 0.00039±0.00010 | 0.00060±0.00003 | 2.74237±0.40846 |
| *CsLEA37* | 0.02215±0.00383 | 0.03307±0.00245 | 0.00268±0.00050 | 0.00429±0.00046 | 0.00168±0.00019 |
| *CsLEA38* | 0.00531±0.00051 | 0.01384±0.00206 | 0.01600±0.00113 | 0.00761±0.00172 | 0.00485±0.00066 |
| *CsLEA39* | 0.00000±0.00000 | 0.00000±0.00000 | 0.00000±0.00000 | 0.00000±0.00000 | 0.01242±0.00280 |
| *CsLEA40* | 0.32141±0.07054 | 0.03647±0.00033 | 0.02442±0.00276 | 0.31340±0.01216 | 0.00740±0.00109 |
| *CsLEA41* | 0.02928±0.00738 | 0.00012±0.00003 | 0.00032±0.00005 | 0.01143±0.00209 | 2.77075±0.60080 |
| *CsLEA42* | 0.10904±0.00219 | 0.05432±0.00885 | 0.09794±0.01275 | 0.20823±0.01092 | 0.02129±0.00338 |
| *CsLEA43* | 0.24913±0.09012 | 0.01671±0.00402 | 0.15371±0.01740 | 0.03140±0.00061 | 0.04973±0.01392 |
| *CsLEA44* | 0.06074±0.00633 | 0.00051±0.00016 | 0.00309±0.00005 | 0.00104±0.00002 | 0.00513±0.00056 |
| *CsLEA45* | 0.08319±0.00647 | 0.00101±0.00023 | 0.01080±0.00133 | 0.00259±0.00012 | 0.00422±0.00089 |
| *CsLEA46* | 0.02266±0.00593 | 0.00080±0.00017 | 0.00203±0.00032 | 0.00023±0.00004 | 0.00481±0.00041 |
| *CsLEA47* | 0.00966±0.00083 | 0.00076±0.00015 | 0.00069±0.00007 | 0.00035±0.00004 | 0.00149±0.00028 |
| *CsLEA48* | 0.32684±0.02099 | 0.02936±0.00392 | 0.04420±0.00348 | 0.01137±0.00072 | 2.90879±0.21486 |

Note: The relative expression values were calculated using the 2^-ΔCt^ method with GAPDH as a housekeeping gene.
