# Supplementary material for: Genome-wide identification and expression analyses of the LEA protein gene family in tea plant reveal their involvement in seed development and abiotic stress responses
Source: Sci Rep. 2019 Oct 1;9:14123. doi: 10.1038/s41598-019-50645-8 (PMC6773783; doi:10.1038/s41598-019-50645-8)
Supplement: Supplementary file 6 — Supplementary Table S5 [file 41598_2019_50645_MOESM6_ESM.docx]

**Genome-wide identification and expression analyses of the LEA protein gene family in tea plant reveal their involvement in seed development and abiotic stress responses**

**Xiaofang Jin^1, 2^, Dan Cao^1^, Zhongjie Wang^2^, Linlong Ma^1^, Kunhong Tian^2^, Yanli Liu^1^, Ziming Gong^1^, Xiangxiang Zhu^2^, Changjun Jiang^2,^ * & Yeyun Li^2,^ ***

^1^ Fruit and Tea Research Institute, Hubei Academy of Agricultural Sciences, Wuhan, 430064, China

^2^ State Key Laboratory of Tea Plant Biology and Utilization, Anhui Agricultural University, Hefei, 230036, China

* Correspondence: jiangcj@ahau.edu.cn; lyy@ahau.edu.cn

**Supplementary Table S5.** The expression levels of 48 *CsLEA* genes during tea seed development process.

| **Gene name** | **Values (Mean ± SD)** | | | | | | |
| --- | --- | --- | --- | --- | --- | --- | --- |
|  | **April** | **May** | **June** | **July** | **August** | **September** | **October** |
| *CsLEA1* | 1.00 | 0.88±0.32 | 0.52±0.10 | 0.42±0.09 | 3.95±0.85 | 1.05±0.12 | 16.26±0.96 |
| *CsLEA2* | 1.00 | 1.03±0.30 | 0.68±0.03 | 1.73±0.16 | 2.70±0.02 | 7.53±0.04 | 52.15±4.09 |
| *CsLEA3* | 1.00 | 1.15±0.17 | 1.08±0.09 | 1.26±0.11 | 1.57±0.01 | 0.53±0.04 | 6.02±0.95 |
| *CsLEA4* | 1.00 | 1.03±0.08 | 0.63±0.05 | 0.67±0.04 | 0.53±0.00 | 0.27±0.05 | 1.54±0.17 |
| *CsLEA5* | 1.00 | 1.19±0.00 | 1.33±0.03 | 1.06±0.10 | 0.42±0.02 | 0.11±0.02 | 5.28±0.31 |
| *CsLEA6* | 1.00 | 0.63±0.07 | 0.87±0.07 | 0.33±0.04 | 0.09±0.02 | 0.11±0.02 | 3.28±0.18 |
| *CsLEA7* | 1.00 | 0.89±0.04 | 0.31±0.03 | 4.65±0.30 | 0.30±0.01 | 0.22±0.05 | 3.85±0.51 |
| *CsLEA8* | 1.00 | 0.59±0.11 | 0.03±0.01 | 0.02±0.00 | 0.04±0.01 | 0.06±0.01 | 1.78±0.15 |
| *CsLEA9* | 1.00 | 0.30±0.08 | 0.05±0.02 | 0.01±0.00 | 0.05±0.01 | 0.01±0.00 | 0.50±0.05 |
| *CsLEA10* | 1.00 | 0.52±0.09 | 0.26±0.04 | 0.11±0.04 | 0.09±0.02 | 0.10±0.03 | 7.66±1.57 |
| *CsLEA11* | 1.00 | 0.22±0.07 | 5.12±1.26 | 100.94±27.13 | 3912.42±317.11 | 4288.66±102.08 | 14112.05±844.43 |
| *CsLEA12* | 1.00 | 0.41±0.09 | 0.18±0.03 | 14.08±4.56 | 520.25±62.81 | 913.98±76.10 | 3302.90±99.28 |
| *CsLEA13* | 1.00 | 1.31±0.15 | 0.82±0.22 | 0.04±0.01 | 0.04±0.01 | 0.07±0.02 | 1.11±0.22 |
| *CsLEA14* | 1.00 | 0.68±0.10 | 0.08±0.02 | 0.32±0.11 | 4.34±1.23 | 20.39±1.08 | 68.32±3.25 |
| *CsLEA15* | 1.00 | 0.77±0.25 | 13.29±2.63 | 68.81±12.60 | 3.41±0.92 | 1.71±0.33 | 9.48±1.26 |
| *CsLEA16* | 1.00 | 0.55±0.02 | 0.34±0.12 | 0.06±0.01 | 0.23±0.00 | 0.30±0.01 | 6.59±0.72 |
| *CsLEA17* | 1.00 | 1.01±0.21 | 0.63±0.17 | 1.03±0.19 | 1.42±0.12 | 1.28±0.15 | 25.29±2.64 |
| *CsLEA18* | 1.00 | 1.41±0.14 | 3.13±0.16 | 2.53±0.02 | 2.73±0.47 | 2.52±0.17 | 14.61±3.67 |
| *CsLEA19* | 1.00 | 0.68±0.13 | 0.33±0.04 | 0.16±0.03 | 0.38±0.08 | 0.70±0.14 | 32.77±1.76 |
| *CsLEA20* | 1.00 | 1.36±0.06 | 1.59±0.19 | 0.37±0.00 | 0.02±0.00 | 0.02±0.00 | 0.38±0.07 |
| *CsLEA21* | 1.00 | 1.80±0.53 | 11.66±2.69 | 0.89±0.20 | 1.21±0.18 | 0.63±0.04 | 1.44±0.11 |
| *CsLEA22* | 1.00 | 1.55±0.44 | 1.28±0.02 | 1.01±0.01 | 1.72±0.09 | 1.95±0.07 | 3.29±0.90 |
| *CsLEA23* | 1.00 | 1.33±0.18 | 0.89±0.18 | 1.72±0.50 | 1.02±0.14 | 2.09±0.49 | 42.01±8.29 |
| *CsLEA24* | 1.00 | 0.93±0.12 | 15.00±0.79 | 0.98±0.03 | 0.47±0.07 | 0.10±0.00 | 4.87±0.52 |
| *CsLEA25* | 1.00 | 2.18±0.05 | 5.53±0.55 | 2.67±0.60 | 2.93±0.54 | 1.43±0.18 | 12.84±1.58 |
| *CsLEA26* | 1.00 | 0.48±0.18 | 0.31±0.07 | 0.09±0.01 | 0.45±0.04 | 0.76±0.22 | 93.20±4.26 |
| *CsLEA27* | 1.00 | 1.15±0.21 | 6.17±0.99 | 5.61±0.34 | 6.13±0.10 | 0.60±0.07 | 3.79±0.76 |
| *CsLEA28* | 1.00 | 0.78±0.24 | 0.72±0.11 | 23.06±3.66 | 467.39±77.60 | 801.81±26.54 | 7767.74±354.63 |
| *CsLEA29* | 1.00 | 1.00±0.27 | 1.42±0.38 | 2.65±0.76 | 1.24±0.14 | 0.56±0.13 | 34.55±4.85 |
| *CsLEA30* | 1.00 | 1.39±0.05 | 1.84±0.01 | 1.51±0.14 | 1.66±0.03 | 0.61±0.04 | 1.24±0.34 |
| *CsLEA31* | 1.00 | 2.11±0.05 | 0.54±0.15 | 0.07±0.00 | 1.87±0.14 | 1.22±0.07 | 2.86±0.30 |
| *CsLEA32* | 1.00 | 1.01±0.01 | 0.19±0.03 | 0.08±0.01 | 0.36±0.01 | 0.64±0.01 | 16.53±3.68 |
| *CsLEA33* | 1.00 | 0.60±0.19 | 0.01±0.00 | 0.01±0.00 | 1.60±0.18 | 11.89±2.32 | 149.23±17.77 |
| *CsLEA34* | 1.00 | 0.47±0.00 | 0.03±0.01 | 0.01±0.00 | 0.48±0.05 | 0.66±0.06 | 9.82±1.50 |
| *CsLEA35* | 1.00 | 3.14±0.23 | 0.88±0.07 | 0.54±0.01 | 0.64±0.03 | 1.14±0.11 | 18.05±0.89 |
| *CsLEA36* | 1.00 | 1.33±0.37 | 0.20±0.02 | 0.70±0.15 | 368.60±9.99 | 836.09±89.12 | 1669.81±114.54 |
| *CsLEA37* | 1.00 | 0.60±0.02 | 1.34±0.19 | 0.06±0.01 | 0.31±0.08 | 0.71±0.00 | 13.79±1.70 |
| *CsLEA38* | 1.00 | 1.42±0.21 | 0.01±0.00 | 0.01±0.00 | 1.71±0.24 | 0.16±0.02 | 2.80±0.23 |
| *CsLEA39* | 1.00 | 2.83±0.92 | 3.19±0.97 | 7.51±1.38 | 3.58±0.79 | 2.77±0.52 | 3631.74±85.64 |
| *CsLEA40* | 1.00 | 3.55±0.34 | 6.40±1.75 | 15.12±2.97 | 31.07±3.72 | 5.36±1.08 | 16.88±4.08 |
| *CsLEA41* | 1.00 | 1.51±0.43 | 12.40±0.90 | 69.22±4.55 | 1393.43±55.32 | 1491.90±54.14 | 6727.30±179.55 |
| *CsLEA42* | 1.00 | 1.89±0.43 | 1.33±0.17 | 0.41±0.04 | 0.65±0.00 | 0.04±0.00 | 1.00±0.28 |
| *CsLEA43* | 1.00 | 1.97±0.56 | 2.17±0.18 | 1.13±0.04 | 6.53±0.18 | 0.10±0.00 | 5.51±0.19 |
| *CsLEA44* | 1.00 | 0.53±0.09 | 0.26±0.10 | 0.20±0.05 | 1.27±0.01 | 0.88±0.06 | 64.17±7.91 |
| *CsLEA45* | 1.00 | 0.46±0.06 | 0.73±0.18 | 0.79±0.13 | 1.85±0.21 | 0.74±0.14 | 39.78±3.68 |
| *CsLEA46* | 1.00 | 1.23±0.20 | 2.48±0.58 | 2.83±0.67 | 3.57±0.59 | 3.37±0.61 | 721.81±11.22 |
| *CsLEA47* | 1.00 | 0.93±0.12 | 0.92±0.24 | 0.94±0.19 | 0.20±0.05 | 0.18±0.03 | 1.00±0.06 |
| *CsLEA48* | 1.00 | 0.54±0.05 | 0.22±0.02 | 2.29±0.14 | 183.35±2.24 | 1677.90±156.39 | 18651.90±911.61 |

Note: The relative expression values were calculated using the 2^-ΔΔCt^ method with GAPDH as a housekeeping gene.
