# Supplementary material for: Genome-wide identification and expression analyses of the LEA protein gene family in tea plant reveal their involvement in seed development and abiotic stress responses
Source: Sci Rep. 2019 Oct 1;9:14123. doi: 10.1038/s41598-019-50645-8 (PMC6773783; doi:10.1038/s41598-019-50645-8)
Supplement: Supplementary file 7 — Supplementary Table S6 [file 41598_2019_50645_MOESM7_ESM.docx]

**Genome-wide identification and expression analyses of the LEA protein gene family in tea plant reveal their involvement in seed development and abiotic stress responses**

**Xiaofang Jin^1, 2^, Dan Cao^1^, Zhongjie Wang^2^, Linlong Ma^1^, Kunhong Tian^2^, Yanli Liu^1^, Ziming Gong^1^, Xiangxiang Zhu^2^, Changjun Jiang^2,^ * & Yeyun Li^2,^ ***

^1^ Fruit and Tea Research Institute, Hubei Academy of Agricultural Sciences, Wuhan, 430064, China

^2^ State Key Laboratory of Tea Plant Biology and Utilization, Anhui Agricultural University, Hefei, 230036, China

* Correspondence: jiangcj@ahau.edu.cn; lyy@ahau.edu.cn

**Supplementary Table S6.** The expression levels of 48 *CsLEA* genes during tea seed desiccation process.

| **Gene name** | **Values (Mean ± SD)** | | | |
| --- | --- | --- | --- | --- |
|  | **0d** | **3d** | **5d** | **8d** |
| *CsLEA1* | 1.00 | 0.92±0.09 | 1.01±0.12 | 0.81±0.15 |
| *CsLEA2* | 1.00 | 1.25±0.06 | 1.22±0.06 | 0.82±0.19 |
| *CsLEA3* | 1.00 | 1.51±0.18 | 1.87±0.22 | 1.27±0.06 |
| *CsLEA4* | 1.00 | 0.51±0.05 | 0.53±0.00 | 0.44±0.06 |
| *CsLEA5* | 1.00 | 0.51±0.03 | 0.45±0.08 | 0.40±0.01 |
| *CsLEA6* | 1.00 | 0.74±0.01 | 0.67±0.16 | 0.75±0.12 |
| *CsLEA7* | 1.00 | 0.40±0.05 | 0.43±0.08 | 3.25±0.15 |
| *CsLEA8* | 1.00 | 0.81±0.06 | 1.19±0.32 | 1.11±0.17 |
| *CsLEA9* | 1.00 | 0.82±0.24 | 0.97±0.13 | 1.24±0.08 |
| *CsLEA10* | 1.00 | 0.84±0.19 | 1.26±0.17 | 0.91±0.23 |
| *CsLEA11* | 1.00 | 0.72±0.02 | 0.44±0.05 | 0.42±0.01 |
| *CsLEA12* | 1.00 | 0.82±0.04 | 0.80±0.12 | 0.72±0.04 |
| *CsLEA13* | 1.00 | 0.27±0.04 | 0.29±0.03 | 0.27±0.01 |
| *CsLEA14* | 1.00 | 1.07±0.06 | 1.21±0.26 | 1.35±0.09 |
| *CsLEA15* | 1.00 | 1.07±0.26 | 1.10±0.38 | 0.71±0.13 |
| *CsLEA16* | 1.00 | 0.49±0.01 | 1.52±0.14 | 1.25±0.09 |
| *CsLEA17* | 1.00 | 0.98±0.26 | 1.66±0.26 | 1.19±0.19 |
| *CsLEA18* | 1.00 | 0.68±0.09 | 0.73±0.07 | 0.61±0.20 |
| *CsLEA19* | 1.00 | 0.57±0.05 | 0.87±0.11 | 0.94±0.09 |
| *CsLEA20* | 1.00 | 1.03±0.17 | 0.89±0.05 | 0.72±0.10 |
| *CsLEA21* | 1.00 | 1.02±0.08 | 1.07±0.25 | 1.02±0.09 |
| *CsLEA22* | 1.00 | 0.69±0.13 | 0.84±0.15 | 0.87±0.13 |
| *CsLEA23* | 1.00 | 3.58±0.94 | 2.81±0.31 | 2.78±0.16 |
| *CsLEA24* | 1.00 | 0.87±0.03 | 1.68±0.12 | 1.69±0.22 |
| *CsLEA25* | 1.00 | 0.61±0.07 | 0.61±0.11 | 0.65±0.17 |
| *CsLEA26* | 1.00 | 0.77±0.06 | 0.73±0.17 | 0.95±0.20 |
| *CsLEA27* | 1.00 | 0.54±0.13 | 0.79±0.08 | 0.81±0.04 |
| *CsLEA28* | 1.00 | 0.97±0.04 | 0.72±0.16 | 1.08±0.11 |
| *CsLEA29* | 1.00 | 1.56±0.34 | 1.17±0.09 | 1.37±0.18 |
| *CsLEA30* | 1.00 | 0.71±0.02 | 0.58±0.08 | 0.68±0.09 |
| *CsLEA31* | 1.00 | 0.54±0.08 | 0.57±0.10 | 0.98±0.05 |
| *CsLEA32* | 1.00 | 0.41±0.02 | 0.46±0.07 | 0.26±0.08 |
| *CsLEA33* | 1.00 | 0.95±0.13 | 1.03±0.02 | 0.86±0.16 |
| *CsLEA34* | 1.00 | 0.79±0.01 | 1.39±0.13 | 1.04±0.23 |
| *CsLEA35* | 1.00 | 1.20±0.34 | 1.77±0.26 | 1.86±0.45 |
| *CsLEA36* | 1.00 | 0.38±0.04 | 0.31±0.03 | 0.32±0.03 |
| *CsLEA37* | 1.00 | 0.91±0.18 | 1.13±0.04 | 1.28±0.13 |
| *CsLEA38* | 1.00 | 1.07±0.22 | 1.54±0.34 | 1.27±0.27 |
| *CsLEA39* | 1.00 | 0.45±0.04 | 0.48±0.02 | 0.72±0.07 |
| *CsLEA40* | 1.00 | 3.09±0.08 | 4.54±0.85 | 2.97±0.12 |
| *CsLEA41* | 1.00 | 0.74±0.01 | 0.58±0.10 | 0.51±0.01 |
| *CsLEA42* | 1.00 | 0.66±0.06 | 0.58±0.06 | 0.51±0.10 |
| *CsLEA43* | 1.00 | 0.09±0.02 | 0.22±0.02 | 0.19±0.02 |
| *CsLEA44* | 1.00 | 0.27±0.03 | 0.43±0.03 | 0.45±0.09 |
| *CsLEA45* | 1.00 | 0.19±0.01 | 0.42±0.04 | 0.35±0.03 |
| *CsLEA46* | 1.00 | 0.46±0.12 | 1.03±0.04 | 0.97±0.12 |
| *CsLEA47* | 1.00 | 1.12±0.24 | 1.56±0.39 | 1.27±0.14 |
| *CsLEA48* | 1.00 | 1.81±0.01 | 1.75±0.28 | 2.59±0.48 |

Note: The relative expression values were calculated using the 2^-ΔΔCt^ method with GAPDH as a housekeeping gene.
