# Supplementary material for: Genome-wide identification and expression analyses of the LEA protein gene family in tea plant reveal their involvement in seed development and abiotic stress responses
Source: Sci Rep. 2019 Oct 1;9:14123. doi: 10.1038/s41598-019-50645-8 (PMC6773783; doi:10.1038/s41598-019-50645-8)
Supplement: Supplementary file 8 — Supplementary Table S7 [file 41598_2019_50645_MOESM8_ESM.docx]

**Genome-wide identification and expression analyses of the LEA protein gene family in tea plant reveal their involvement in seed development and abiotic stress responses**

**Xiaofang Jin^1, 2^, Dan Cao^1^, Zhongjie Wang^2^, Linlong Ma^1^, Kunhong Tian^2^, Yanli Liu^1^, Ziming Gong^1^, Xiangxiang Zhu^2^, Changjun Jiang^2,^ * & Yeyun Li^2,^ ***

^1^ Fruit and Tea Research Institute, Hubei Academy of Agricultural Sciences, Wuhan, 430064, China

^2^ State Key Laboratory of Tea Plant Biology and Utilization, Anhui Agricultural University, Hefei, 230036, China

* Correspondence: jiangcj@ahau.edu.cn; lyy@ahau.edu.cn

**Supplementary Table S7.** The expression levels of 47 *CsLEA* genes in response to low temperature stress.

| **Gene name** | **Values (Mean ± SD)** | | | |
| --- | --- | --- | --- | --- |
|  | **0h** | **6h** | **12h** | **24h** |
| *CsLEA1* | 1.00 | 1.79±0.27 | 1.81±0.16 | 3.06±0.14 |
| *CsLEA2* | 1.00 | 1.02±0.14 | 0.91±0.30 | 1.30±0.23 |
| *CsLEA3* | 1.00 | 1.08±0.33 | 0.87±0.02 | 1.02±0.12 |
| *CsLEA4* | 1.00 | 1.18±0.11 | 1.09±0.03 | 1.30±0.01 |
| *CsLEA5* | 1.00 | 1.88±0.00 | 1.97±0.19 | 2.72±0.73 |
| *CsLEA6* | 1.00 | 0.75±0.14 | 0.67±0.10 | 1.40±0.44 |
| *CsLEA7* | 1.00 | 1.19±0.14 | 0.93±0.03 | 1.16±0.22 |
| *CsLEA8* | 1.00 | 0.70±0.13 | 0.68±0.13 | 1.53±0.18 |
| *CsLEA9* | 1.00 | 1.21±0.11 | 0.85±0.05 | 1.38±0.06 |
| *CsLEA10* | 1.00 | 1.36±0.08 | 0.60±0.14 | 1.06±0.20 |
| *CsLEA11* | 1.00 | 1.47±0.26 | 1.87±0.27 | 1.34±0.19 |
| *CsLEA12* | 1.00 | 1.72±0.25 | 1.62±0.27 | 7.28±0.65 |
| *CsLEA13* | 1.00 | 2.87±0.16 | 2.25±0.14 | 6.46±0.57 |
| *CsLEA14* | 1.00 | 0.91±0.24 | 0.57±0.07 | 1.37±0.20 |
| *CsLEA15* | 1.00 | 0.87±0.08 | 0.47±0.08 | 1.04±0.11 |
| *CsLEA16* | 1.00 | 0.33±0.09 | 0.20±0.05 | 0.76±0.10 |
| *CsLEA17* | 1.00 | 0.83±0.10 | 0.75±0.08 | 0.76±0.13 |
| *CsLEA18* | 1.00 | 1.14±0.03 | 0.97±0.02 | 1.24±0.36 |
| *CsLEA19* | 1.00 | 0.86±0.15 | 1.25±0.23 | 2.60±0.39 |
| *CsLEA20* | 1.00 | 1.23±0.13 | 0.82±0.21 | 1.66±0.12 |
| *CsLEA21* | 1.00 | 6.12±0.34 | 4.91±0.20 | 3.87±0.46 |
| *CsLEA22* | 1.00 | 2.07±0.09 | 2.43±0.41 | 1.55±0.35 |
| *CsLEA23* | 1.00 | 1.12±0.16 | 0.98±0.08 | 1.54±0.22 |
| *CsLEA24* | 1.00 | 21.17±2.34 | 13.18±1.72 | 7.56±1.80 |
| *CsLEA25* | 1.00 | 0.65±0.09 | 0.52±0.04 | 0.81±0.11 |
| *CsLEA26* | 1.00 | 0.95±0.03 | 0.70±0.07 | 2.08±0.23 |
| *CsLEA27* | 1.00 | 1.22±0.23 | 1.64±0.36 | 3.01±0.32 |
| *CsLEA28* | 1.00 | 0.63±0.07 | 0.46±0.01 | 1.00±0.06 |
| *CsLEA29* | 1.00 | 0.57±0.09 | 0.77±0.23 | 0.95±0.15 |
| *CsLEA30* | 1.00 | 1.19±0.37 | 1.17±0.14 | 2.01±0.27 |
| *CsLEA31* | 1.00 | 1.88±0.57 | 1.63±0.27 | 3.29±0.91 |
| *CsLEA32* | 1.00 | 5.54±0.75 | 6.76±1.00 | 22.35±3.02 |
| *CsLEA33* | 1.00 | 0.80±0.06 | 0.86±0.02 | 1.62±0.57 |
| *CsLEA34* | 1.00 | 0.44±0.12 | 0.42±0.13 | 1.32±0.32 |
| *CsLEA35* | 1.00 | 0.56±0.01 | 0.65±0.03 | 0.96±0.05 |
| *CsLEA36* | 1.00 | 0.36±0.05 | 0.65±0.08 | 1.03±0.16 |
| *CsLEA37* | 1.00 | 0.76±0.16 | 1.46±0.11 | 1.47±0.09 |
| *CsLEA38* | 1.00 | 1.39±0.26 | 0.90±0.01 | 1.45±0.48 |
| *CsLEA40* | 1.00 | 1.04±0.09 | 1.01±0.10 | 1.40±0.28 |
| *CsLEA41* | 1.00 | 0.59±0.11 | 0.55±0.06 | 1.47±0.23 |
| *CsLEA42* | 1.00 | 1.28±0.08 | 1.67±0.33 | 2.30±0.08 |
| *CsLEA43* | 1.00 | 2.01±0.14 | 1.07±0.24 | 0.99±0.14 |
| *CsLEA44* | 1.00 | 1.03±0.37 | 1.00±0.29 | 1.89±0.32 |
| *CsLEA45* | 1.00 | 3.29±0.17 | 2.57±0.53 | 8.85±0.17 |
| *CsLEA46* | 1.00 | 1.02±0.15 | 1.00±0.12 | 1.95±0.26 |
| *CsLEA47* | 1.00 | 0.56±0.02 | 0.60±0.08 | 0.39±0.09 |
| *CsLEA48* | 1.00 | 0.56±0.11 | 0.84±0.04 | 2.15±0.06 |

Note: The relative expression values were calculated using the 2^-ΔΔCt^ method with GAPDH as a housekeeping gene.
