# Supplementary material for: Genome-wide identification and expression analyses of the LEA protein gene family in tea plant reveal their involvement in seed development and abiotic stress responses
Source: Sci Rep. 2019 Oct 1;9:14123. doi: 10.1038/s41598-019-50645-8 (PMC6773783; doi:10.1038/s41598-019-50645-8)
Supplement: Supplementary file 9 — Supplementary Table S8 [file 41598_2019_50645_MOESM9_ESM.docx]

**Genome-wide identification and expression analyses of the LEA protein gene family in tea plant reveal their involvement in seed development and abiotic stress responses**

**Xiaofang Jin^1, 2^, Dan Cao^1^, Zhongjie Wang^2^, Linlong Ma^1^, Kunhong Tian^2^, Yanli Liu^1^, Ziming Gong^1^, Xiangxiang Zhu^2^, Changjun Jiang^2,^ * & Yeyun Li^2,^ ***

^1^ Fruit and Tea Research Institute, Hubei Academy of Agricultural Sciences, Wuhan, 430064, China

^2^ State Key Laboratory of Tea Plant Biology and Utilization, Anhui Agricultural University, Hefei, 230036, China

* Correspondence: jiangcj@ahau.edu.cn; lyy@ahau.edu.cn

**Supplementary Table S8.** The expression levels of 47 *CsLEA* genes in response to high temperature stress.

| **Gene name** | **Values (Mean ± SD)** | | | |
| --- | --- | --- | --- | --- |
|  | **0h** | **6h** | **12h** | **24h** |
| *CsLEA1* | 1.00 | 0.82±0.18 | 2.15±0.18 | 3.68±0.24 |
| *CsLEA2* | 1.00 | 0.72±0.06 | 2.09±0.09 | 2.84±0.27 |
| *CsLEA3* | 1.00 | 1.25±0.15 | 3.87±0.57 | 4.21±0.57 |
| *CsLEA4* | 1.00 | 1.22±0.11 | 3.03±1.01 | 3.36±0.45 |
| *CsLEA5* | 1.00 | 1.38±0.27 | 2.93±0.26 | 4.09±0.28 |
| *CsLEA6* | 1.00 | 0.52±0.09 | 1.72±0.37 | 3.27±0.13 |
| *CsLEA7* | 1.00 | 0.53±0.20 | 1.78±0.17 | 3.31±1.09 |
| *CsLEA8* | 1.00 | 0.67±0.12 | 2.36±0.15 | 4.15±0.38 |
| *CsLEA9* | 1.00 | 0.80±0.10 | 3.34±0.33 | 5.63±0.95 |
| *CsLEA10* | 1.00 | 0.87±0.16 | 4.06±0.59 | 6.00±0.32 |
| *CsLEA11* | 1.00 | 2.11±0.21 | 3.91±0.66 | 7.64±0.25 |
| *CsLEA12* | 1.00 | 1.41±0.13 | 3.33±0.14 | 7.87±1.36 |
| *CsLEA13* | 1.00 | 0.90±0.04 | 1.84±0.12 | 3.81±0.70 |
| *CsLEA14* | 1.00 | 0.72±0.01 | 1.65±0.51 | 5.03±0.71 |
| *CsLEA15* | 1.00 | 1.03±0.10 | 2.52±0.30 | 6.08±0.32 |
| *CsLEA16* | 1.00 | 0.70±0.15 | 1.84±0.57 | 3.37±0.10 |
| *CsLEA17* | 1.00 | 0.69±0.13 | 0.71±0.03 | 1.99±0.64 |
| *CsLEA18* | 1.00 | 5.63±0.39 | 8.20±0.21 | 14.44±1.25 |
| *CsLEA19* | 1.00 | 0.57±0.07 | 0.60±0.07 | 3.88±0.43 |
| *CsLEA20* | 1.00 | 0.81±0.08 | 1.35±0.13 | 2.28±0.77 |
| *CsLEA21* | 1.00 | 3.01±0.27 | 5.42±0.35 | 10.36±1.78 |
| *CsLEA22* | 1.00 | 1.89±0.19 | 1.72±0.23 | 3.99±0.43 |
| *CsLEA23* | 1.00 | 1.99±0.24 | 1.23±0.21 | 3.30±0.69 |
| *CsLEA24* | 1.00 | 1.31±0.15 | 2.61±0.59 | 4.87±0.11 |
| *CsLEA25* | 1.00 | 1.27±0.07 | 2.18±0.36 | 3.66±0.42 |
| *CsLEA26* | 1.00 | 0.99±0.16 | 2.69±0.24 | 3.08±1.06 |
| *CsLEA27* | 1.00 | 1.97±0.11 | 2.55±0.18 | 3.37±0.39 |
| *CsLEA28* | 1.00 | 3.74±0.54 | 4.35±0.85 | 9.85±0.76 |
| *CsLEA29* | 1.00 | 0.94±0.25 | 2.21±0.59 | 3.28±0.19 |
| *CsLEA30* | 1.00 | 1.07±0.30 | 1.54±0.14 | 2.45±0.28 |
| *CsLEA31* | 1.00 | 0.82±0.04 | 1.74±0.15 | 2.70±0.19 |
| *CsLEA32* | 1.00 | 0.85±0.07 | 0.91±0.22 | 1.22±0.14 |
| *CsLEA33* | 1.00 | 2.35±0.79 | 2.41±0.14 | 5.10±0.68 |
| *CsLEA34* | 1.00 | 0.53±0.11 | 1.86±0.08 | 5.62±0.84 |
| *CsLEA35* | 1.00 | 0.71±0.08 | 2.77±0.52 | 3.05±0.20 |
| *CsLEA36* | 1.00 | 0.83±0.06 | 2.42±0.24 | 3.25±0.19 |
| *CsLEA37* | 1.00 | 0.79±0.12 | 1.42±0.39 | 3.70±0.07 |
| *CsLEA38* | 1.00 | 0.73±0.03 | 0.92±0.27 | 2.04±0.60 |
| *CsLEA40* | 1.00 | 2.10±0.40 | 3.74±0.09 | 3.75±0.46 |
| *CsLEA41* | 1.00 | 1.24±0.15 | 4.25±0.66 | 7.03±0.73 |
| *CsLEA42* | 1.00 | 0.47±0.02 | 1.12±0.23 | 1.69±0.30 |
| *CsLEA43* | 1.00 | 0.62±0.11 | 0.92±0.28 | 1.27±0.09 |
| *CsLEA44* | 1.00 | 0.79±0.10 | 2.69±0.05 | 2.41±0.44 |
| *CsLEA45* | 1.00 | 0.60±0.01 | 2.46±0.66 | 2.24±0.62 |
| *CsLEA46* | 1.00 | 1.23±0.17 | 2.74±0.08 | 2.24±0.11 |
| *CsLEA47* | 1.00 | 2.99±0.63 | 3.74±0.27 | 4.55±0.42 |
| *CsLEA48* | 1.00 | 1.00±0.09 | 0.48±0.11 | 1.40±0.16 |

Note: The relative expression values were calculated using the 2^-ΔΔCt^ method with GAPDH as a housekeeping gene.
