# Supplementary material for: Genome-wide identification and expression analyses of the LEA protein gene family in tea plant reveal their involvement in seed development and abiotic stress responses
Source: Sci Rep. 2019 Oct 1;9:14123. doi: 10.1038/s41598-019-50645-8 (PMC6773783; doi:10.1038/s41598-019-50645-8)
Supplement: Supplementary file 10 — Supplementary Table S9 [file 41598_2019_50645_MOESM10_ESM.docx]

**Genome-wide identification and expression analyses of the LEA protein gene family in tea plant reveal their involvement in seed development and abiotic stress responses**

**Xiaofang Jin^1, 2^, Dan Cao^1^, Zhongjie Wang^2^, Linlong Ma^1^, Kunhong Tian^2^, Yanli Liu^1^, Ziming Gong^1^, Xiangxiang Zhu^2^, Changjun Jiang^2,^ * & Yeyun Li^2,^ ***

^1^ Fruit and Tea Research Institute, Hubei Academy of Agricultural Sciences, Wuhan, 430064, China

^2^ State Key Laboratory of Tea Plant Biology and Utilization, Anhui Agricultural University, Hefei, 230036, China

* Correspondence: jiangcj@ahau.edu.cn; lyy@ahau.edu.cn

**Supplementary Table S9.** The expression levels of 47 *CsLEA* genes in response to drought stress.

| **Gene name** | **Values (Mean ± SD)** | | | |
| --- | --- | --- | --- | --- |
|  | **0h** | **6h** | **12h** | **24h** |
| *CsLEA1* | 1.00 | 0.75±0.08 | 1.49±0.07 | 1.80±0.22 |
| *CsLEA2* | 1.00 | 0.67±0.13 | 0.64±0.13 | 0.45±0.09 |
| *CsLEA3* | 1.00 | 2.15±0.22 | 3.09±0.54 | 2.30±0.25 |
| *CsLEA4* | 1.00 | 0.95±0.07 | 2.05±0.72 | 2.37±0.17 |
| *CsLEA5* | 1.00 | 2.00±0.26 | 3.23±0.55 | 1.97±0.43 |
| *CsLEA6* | 1.00 | 1.09±0.03 | 2.22±0.38 | 1.78±0.07 |
| *CsLEA7* | 1.00 | 0.99±0.17 | 1.93±0.19 | 1.30±0.16 |
| *CsLEA8* | 1.00 | 0.68±0.04 | 1.63±0.00 | 1.03±0.14 |
| *CsLEA9* | 1.00 | 0.59±0.21 | 1.22±0.26 | 0.83±0.03 |
| *CsLEA10* | 1.00 | 0.64±0.06 | 1.52±0.28 | 0.97±0.33 |
| *CsLEA11* | 1.00 | 3.79±0.07 | 7.08±0.47 | 5.57±0.36 |
| *CsLEA12* | 1.00 | 0.54±0.02 | 1.16±0.26 | 0.87±0.07 |
| *CsLEA13* | 1.00 | 0.98±0.19 | 1.97±0.13 | 1.61±0.28 |
| *CsLEA14* | 1.00 | 2.50±0.22 | 5.61±0.62 | 7.73±0.99 |
| *CsLEA15* | 1.00 | 1.85±0.12 | 1.67±0.33 | 2.36±0.26 |
| *CsLEA16* | 1.00 | 1.06±0.06 | 2.60±0.18 | 3.11±0.21 |
| *CsLEA17* | 1.00 | 0.98±0.11 | 0.84±0.07 | 0.77±0.25 |
| *CsLEA18* | 1.00 | 1.89±0.14 | 2.29±0.30 | 8.27±0.49 |
| *CsLEA19* | 1.00 | 1.44±0.25 | 1.97±0.08 | 4.53±0.24 |
| *CsLEA20* | 1.00 | 1.21±0.14 | 1.51±0.40 | 3.45±0.15 |
| *CsLEA21* | 1.00 | 0.87±0.03 | 1.44±0.08 | 3.20±0.46 |
| *CsLEA22* | 1.00 | 0.76±0.15 | 0.88±0.11 | 1.31±0.19 |
| *CsLEA23* | 1.00 | 0.65±0.05 | 0.60±0.03 | 0.56±0.13 |
| *CsLEA24* | 1.00 | 0.52±0.11 | 0.78±0.06 | 0.94±0.00 |
| *CsLEA25* | 1.00 | 0.94±0.13 | 0.79±0.17 | 1.36±0.08 |
| *CsLEA26* | 1.00 | 0.67±0.07 | 1.41±0.28 | 1.99±0.10 |
| *CsLEA27* | 1.00 | 2.95±0.22 | 0.60±0.04 | 0.85±0.18 |
| *CsLEA28* | 1.00 | 5.95±0.48 | 6.40±0.22 | 11.24±1.95 |
| *CsLEA29* | 1.00 | 0.48±0.14 | 0.42±0.11 | 0.92±0.29 |
| *CsLEA30* | 1.00 | 1.69±0.09 | 3.20±0.19 | 3.20±0.71 |
| *CsLEA31* | 1.00 | 0.05±0.01 | 0.17±0.06 | 0.57±0.02 |
| *CsLEA32* | 1.00 | 3.10±0.03 | 3.19±0.60 | 2.85±0.14 |
| *CsLEA33* | 1.00 | 1.75±0.11 | 2.69±0.88 | 5.23±0.45 |
| *CsLEA34* | 1.00 | 0.70±0.18 | 2.14±0.05 | 1.75±0.17 |
| *CsLEA35* | 1.00 | 0.32±0.00 | 0.33±0.06 | 0.18±0.01 |
| *CsLEA36* | 1.00 | 13.82±1.14 | 17.56±1.53 | 6.59±0.22 |
| *CsLEA37* | 1.00 | 0.42±0.08 | 2.08±0.17 | 0.95±0.23 |
| *CsLEA38* | 1.00 | 0.56±0.17 | 0.77±0.04 | 3.43±0.21 |
| *CsLEA40* | 1.00 | 1.12±0.16 | 2.04±0.11 | 4.03±0.38 |
| *CsLEA41* | 1.00 | 0.71±0.14 | 2.07±0.16 | 3.08±0.50 |
| *CsLEA42* | 1.00 | 0.45±0.00 | 0.54±0.01 | 0.89±0.08 |
| *CsLEA43* | 1.00 | 0.31±0.00 | 0.39±0.02 | 0.92±0.07 |
| *CsLEA44* | 1.00 | 0.28±0.03 | 0.84±0.06 | 5.11±1.16 |
| *CsLEA45* | 1.00 | 0.61±0.01 | 1.59±0.19 | 10.45±3.09 |
| *CsLEA46* | 1.00 | 0.75±0.13 | 0.29±0.02 | 0.61±0.06 |
| *CsLEA47* | 1.00 | 1.53±0.15 | 1.39±0.14 | 0.97±0.13 |
| *CsLEA48* | 1.00 | 3.86±0.09 | 7.69±0.23 | 7.90±0.32 |

Note: The relative expression values were calculated using the 2^-ΔΔCt^ method with GAPDH as a housekeeping gene.
