# Supplementary material for: Genome-wide identification and expression analyses of the LEA protein gene family in tea plant reveal their involvement in seed development and abiotic stress responses
Source: Sci Rep. 2019 Oct 1;9:14123. doi: 10.1038/s41598-019-50645-8 (PMC6773783; doi:10.1038/s41598-019-50645-8)
Supplement: Supplementary file 11 — Supplementary Table S10 [file 41598_2019_50645_MOESM11_ESM.docx]

**Genome-wide identification and expression analyses of the LEA protein gene family in tea plant reveal their involvement in seed development and abiotic stress responses**

**Xiaofang Jin^1, 2^, Dan Cao^1^, Zhongjie Wang^2^, Linlong Ma^1^, Kunhong Tian^2^, Yanli Liu^1^, Ziming Gong^1^, Xiangxiang Zhu^2^, Changjun Jiang^2,^ * & Yeyun Li^2,^ ***

^1^ Fruit and Tea Research Institute, Hubei Academy of Agricultural Sciences, Wuhan, 430064, China

^2^ State Key Laboratory of Tea Plant Biology and Utilization, Anhui Agricultural University, Hefei, 230036, China

* Correspondence: jiangcj@ahau.edu.cn; lyy@ahau.edu.cn

**Supplementary Table S10.** The expression levels of 47 *CsLEA* genes in response to ABA stress.

| **Gene name** | **Values (Mean ± SD)** | | | |
| --- | --- | --- | --- | --- |
|  | **0h** | **6h** | **12h** | **24h** |
| *CsLEA1* | 1.00 | 0.87±0.18 | 1.35±0.27 | 1.53±0.41 |
| *CsLEA2* | 1.00 | 0.59±0.06 | 0.93±0.09 | 1.06±0.20 |
| *CsLEA3* | 1.00 | 1.83±0.15 | 1.85±0.14 | 2.30±0.01 |
| *CsLEA4* | 1.00 | 0.72±0.01 | 0.72±0.03 | 0.79±0.03 |
| *CsLEA5* | 1.00 | 1.30±0.13 | 0.82±0.03 | 0.99±0.01 |
| *CsLEA6* | 1.00 | 0.99±0.04 | 1.36±0.38 | 1.33±0.04 |
| *CsLEA7* | 1.00 | 0.71±0.01 | 0.75±0.12 | 0.69±0.12 |
| *CsLEA8* | 1.00 | 2.10±0.27 | 3.58±0.40 | 4.77±0.73 |
| *CsLEA9* | 1.00 | 0.87±0.01 | 1.27±0.08 | 3.22±0.58 |
| *CsLEA10* | 1.00 | 2.48±0.50 | 3.47±0.04 | 4.48±0.21 |
| *CsLEA11* | 1.00 | 0.34±0.11 | 0.66±0.15 | 0.64±0.07 |
| *CsLEA12* | 1.00 | 1.84±0.10 | 1.71±0.52 | 2.62±0.59 |
| *CsLEA13* | 1.00 | 0.51±0.09 | 0.47±0.13 | 0.51±0.02 |
| *CsLEA14* | 1.00 | 2.38±0.39 | 4.59±0.34 | 5.06±0.84 |
| *CsLEA15* | 1.00 | 0.47±0.07 | 0.83±0.10 | 0.83±0.01 |
| *CsLEA16* | 1.00 | 1.59±0.08 | 2.00±0.58 | 2.34±0.39 |
| *CsLEA17* | 1.00 | 1.04±0.09 | 1.10±0.05 | 0.89±0.20 |
| *CsLEA18* | 1.00 | 1.04±0.07 | 1.01±0.17 | 1.10±0.06 |
| *CsLEA19* | 1.00 | 3.23±1.06 | 3.28±0.35 | 4.41±0.97 |
| *CsLEA20* | 1.00 | 0.63±0.00 | 0.52±0.05 | 0.70±0.05 |
| *CsLEA21* | 1.00 | 0.49±0.05 | 0.51±0.04 | 0.49±0.08 |
| *CsLEA22* | 1.00 | 0.34±0.04 | 0.29±0.07 | 0.32±0.05 |
| *CsLEA23* | 1.00 | 0.91±0.22 | 0.80±0.10 | 1.19±0.10 |
| *CsLEA24* | 1.00 | 0.29±0.01 | 0.43±0.05 | 0.57±0.02 |
| *CsLEA25* | 1.00 | 1.62±0.28 | 1.30±0.11 | 1.65±0.00 |
| *CsLEA26* | 1.00 | 3.04±0.46 | 4.69±0.31 | 6.28±0.19 |
| *CsLEA27* | 1.00 | 2.99±0.68 | 2.93±0.74 | 1.98±0.03 |
| *CsLEA28* | 1.00 | 0.49±0.02 | 0.54±0.01 | 0.47±0.08 |
| *CsLEA29* | 1.00 | 0.75±0.18 | 1.28±0.17 | 1.24±0.29 |
| *CsLEA30* | 1.00 | 1.45±0.39 | 1.31±0.10 | 1.35±0.12 |
| *CsLEA31* | 1.00 | 0.11±0.02 | 0.16±0.02 | 0.11±0.01 |
| *CsLEA32* | 1.00 | 1.43±0.02 | 1.19±0.06 | 0.74±0.09 |
| *CsLEA33* | 1.00 | 1.31±0.01 | 2.32±0.72 | 2.07±0.24 |
| *CsLEA34* | 1.00 | 4.43±0.85 | 9.73±1.01 | 6.24±0.17 |
| *CsLEA35* | 1.00 | 1.72±0.36 | 3.27±0.21 | 1.57±0.45 |
| *CsLEA36* | 1.00 | 2.82±0.81 | 8.08±0.04 | 2.49±0.67 |
| *CsLEA37* | 1.00 | 3.61±0.34 | 8.54±0.83 | 1.60±0.08 |
| *CsLEA38* | 1.00 | 1.23±0.20 | 2.51±0.36 | 1.33±0.14 |
| *CsLEA40* | 1.00 | 0.73±0.08 | 0.98±0.06 | 0.67±0.07 |
| *CsLEA41* | 1.00 | 4.56±0.20 | 5.91±1.00 | 12.17±0.46 |
| *CsLEA42* | 1.00 | 0.38±0.01 | 0.68±0.05 | 1.00±0.02 |
| *CsLEA43* | 1.00 | 0.61±0.03 | 0.51±0.04 | 0.66±0.01 |
| *CsLEA44* | 1.00 | 0.46±0.07 | 0.99±0.12 | 0.95±0.02 |
| *CsLEA45* | 1.00 | 1.25±0.12 | 0.85±0.07 | 1.05±0.09 |
| *CsLEA46* | 1.00 | 0.68±0.21 | 0.86±0.18 | 0.89±0.05 |
| *CsLEA47* | 1.00 | 1.35±0.09 | 1.07±0.13 | 1.21±0.27 |
| *CsLEA48* | 1.00 | 0.88±0.13 | 4.44±0.21 | 0.70±0.13 |

Note: The relative expression values were calculated using the 2^-ΔΔCt^ method with GAPDH as a housekeeping gene.
